# Assessment of the Purity of IMM-H014 and Its Related Substances for the Treatment of Metabolic-Associated Fatty Liver Disease Using Quantitative Nuclear Magnetic Resonance Spectroscopy

**DOI:** 10.3390/ijms242417508

**Published:** 2023-12-15

**Authors:** Hanyilan Zhang, Haowen Zhu, Song Wu, Haoyang Tang, Wenxuan Zhang, Xiaoliang Gong, Tiesong Wang, Yinghong Wang, Qingyun Yang

**Affiliations:** 1 State Key Laboratory of Bioactive Substance and Function of Natural Medicines, Institute of Materia Medica, Chinese Academy of Medical Sciences & Peking Union Medical College, Beijing 100050, China; zhanghanyilan@imm.ac.cn (H.Z.); zhuhaowen@imm.ac.cn (H.Z.); ws@imm.ac.cn (S.W.); tanghaoyang2023@outlook.com (H.T.); wxzhang@imm.ac.cn (W.Z.); gongxiaoliang@imm.ac.cn (X.G.); 2 NMPA Key Laboratory for Research and Evaluation of Generic Drugs, Beijing Institute for Drug Control, Beijing 102206, China; vip13255893486@163.com

**Keywords:** IMM-H014, quantitative proton nuclear magnetic resonance, methodology validation, purity, metabolic-associated fatty liver disease

## Abstract

An accurate, rapid, and selective quantitative nuclear magnetic resonance method was developed and validated to assess the purity of IMM-H014, a novel drug for the treatment of metabolic-associated fatty liver disease (MAFLD), and four related substances (impurities I, II, III, and IV). In this study, we obtained spectra of IMM--H014 and related substances in deuterated chloroform using dimethyl terephthalate (DMT) as the internal standard reference. Quantification was performed using the ^1^H resonance signals at *δ* 8.13 ppm for DMT and *δ* 6.5–7.5 ppm for IMM-H014 and its related substances. Several key experimental parameters were investigated and optimized, such as pulse angle and relaxation delay. Methodology validation was conducted based on the International Council for Harmonization guidelines and verified with satisfactory specificity, precision, linearity, accuracy, robustness, and stability. In addition, the calibration results of the samples were consistent with those obtained from the mass balance method. Thus, this research provides a reliable and practical protocol for purity analysis of IMM-H014 and its critical impurities and contributes to subsequent clinical quality control research.

## 1. Introduction

Metabolic-associated fatty liver disease (MAFLD), which used to be called non-alcoholic fatty lipid disease, is a hepatic manifestation of a metabolic syndrome characterized by hepatocellular lipid degeneration and fat accumulation and has been recognized as the most prevalent chronic disease [1,2,3], with a consistently high prevalence in several countries and regions, including China [4]. Furthermore, an increasing number of adolescents are being diagnosed with MAFLD, with an alarming prevalence of up to 18% [5]. Even worse, MAFLD encompasses a spectrum of liver tissue pathologies ranging from metabolic-associated fatty liver (MAFL) to metabolic-associated steatohepatitis [5], the latter of which has the potential to progress to cirrhosis and hepatocellular carcinoma [6,7], posing a significant threat to life. Nonetheless, there are still no approved definitive therapeutic agents, and treatments are mainly lifestyle modifications and dietary interventions [8], such as physical activity and caloric restriction [9]. However, lifestyle interventions are not implemented consistently and effectively in most cases. Vitamin E and pioglitazone, used as the first-line medication, have become therapeutic strategies and disease management approaches for patients [10]. However, there are concerns about the long-term safety of vitamin E [11,12]. As a result, the market demand for drugs for treating MAFLD has increased in recent years.

To address the problem, our team successfully synthesized methyl 7,7′-dimethoxy-5-(morpholinomethyl)-[4,4′-bibenzo[d] [1,3] dioxole-5-carboxylate methanesulfonate (codenamed IMM-H014) based on the bicyclol of structure [13]. Bicyclol was approved for the treatment of chronic hepatitis in China in 2004, and it has been shown to have significant inhibitory effects on oxidative stress and to prevent hepatocellular injury [14,15]. According to our preclinical studies of IMM-H014 [16], it has better pharmacokinetic properties and pharmacological activities for liver protection than bicyclol. In addition, the dosage form of IMM-H014 extended-release tablets has been successfully developed [17]. Therefore, IMM-H014 has excellent potential to be an effective drug for treating MAFLD, and the extended-release tablets are currently in phase I clinical trials.

To ensure the safety and efficacy of this new drug, our research group has performed preliminary quality control research. For example, a selective high-performance liquid chromatography (HPLC) method and a sensitive liquid chromatography–tandem mass spectrometry (LC-MS/MS) method have been developed and validated for quantitative analysis of impurities [18]. A novel derivatization HPLC method has also been developed for genotoxic impurities [19]. Reference materials (RMs) for active pharmaceutical ingredients (APIs) and their impurities at an accurate and reliable purity are essential for quality control studies. Thus, it is necessary to establish methods for determining the absolute purity of APIs and their impurities.

Nuclear magnetic resonance (NMR) spectroscopy has shown great potential for quantitative analysis, as the peak area is directly correlated with the number of nuclei [20,21], allowing accurate determination of the absolute purity of organic compounds. Quantitative NMR (qNMR) has emerged as an alternative to the mass balance method, which enables the determination of purity directly upon comparison with an internal standard (IS) [22]. The absolute quantitative method qNMR has been accepted by the International Conference on Harmonization (ICH) and Pharmacopoeia for calibrating the purity of APIs and their related substances [23] because of its multiple advantages over relative quantitative methods such as LC-MS, including simple sample preparation, short analytical time, and no requirement for RMs [24,25].

In this study, we developed and validated a new, accurate, and reliable ^1^H-qNMR method to accurately calibrate the absolute purity of IMM-H014 and its impurities I–IV. The proposed method involves an inexpensive commercially available chemical reagent, dimethyl terephthalate (DMT), as the quantification IS, trimethylsilane (TMS) as the IS of the chemical shift (at *δ* 0 ppm), and deuterated chloroform (CDCl_3_) as the diluent. Several crucial experimental parameters were explored and optimized. In addition, the qNMR method was systematically validated against ICH Q2(R1) to confirm its specificity, robustness, linearity, range, precision, accuracy, and solution stability [26], and the calibration results were compared with those obtained using the mass balance method. The results demonstrated that the proposed method for the purity analysis of IMM-H014 and its critical impurities was accurate, selective, precise, and linear. It provides reliable technical support for the subsequent use of RMs in clinical and non-clinical studies.

## 2. Results and Discussion

### 2.1. Proton Signal Assignments

**Figure 1:**
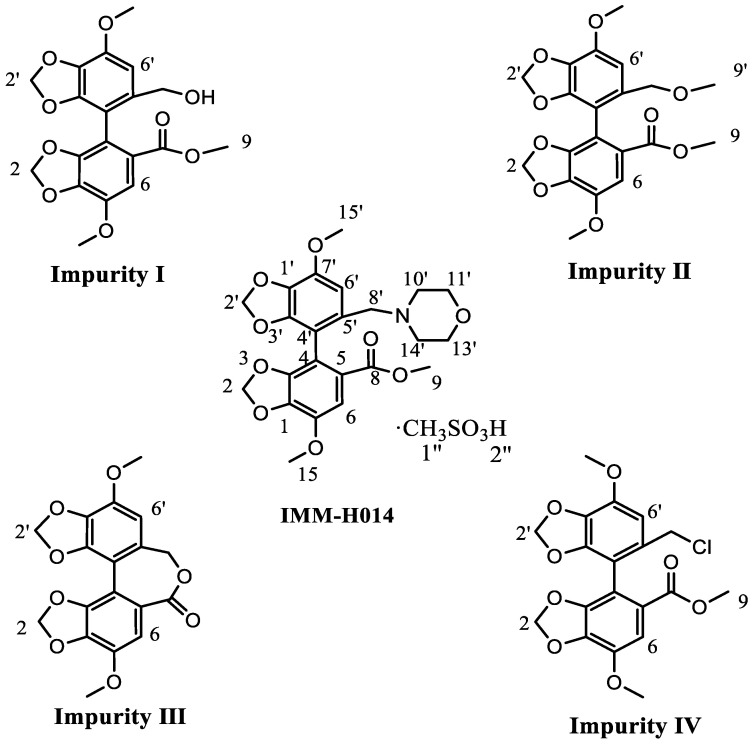
Chemical structures of IMM-H014 and its related substances (impurities I–IV).

In our previous study [18], IMM-H014 and its major impurities were identified, namely, the starting materials (impurity I), reaction byproducts (impurities II and III), and intermediates (impurity IV). Figure 1 displays their chemical structures, and the Supplementary Materials Figures S1–S5 show the detailed spectral data. In the ^1^H-NMR spectra, the obtained proton signals were assigned for the characterized compounds, as listed in Table 1.

### 2.2. Selection of Quantitative Signal and Internal Standard

**Figure 2:**
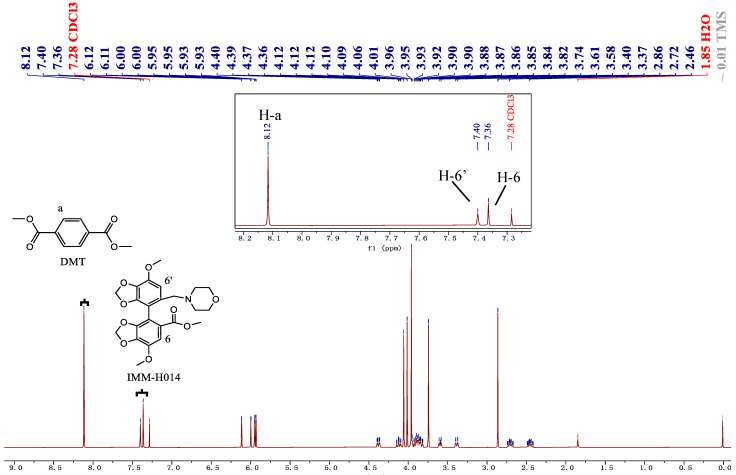
Spectra of the sample of IS and IMM-H014 and enlarged view in downfield.

The key to the qNMR method is to select quantitative signals from analytes. Appropriate quantification signals should meet several criteria, including good separation from other signals and line shapes. Generally, protons from the analyte skeleton are preferred as quantitative protons because they are constant, have less interference, and have better commonality [27]. In addition, quantitative signals should not be selected for complicated and overlapping multiplets upfield. Based on this reasoning, combined with the chemical shift values in Table 1 and Figure 2, the ^1^H-NMR spectra of IMM-H014 and its impurities showed the characteristic signal at approximately *δ* 7.4 ppm (1H, *s*, 6′-H), which belongs to the hydrogen proton on the phenyl group (C-6′). This signal presents a good line shape (single peak) due to the lack of protons coupled with it.

**Figure 3:**
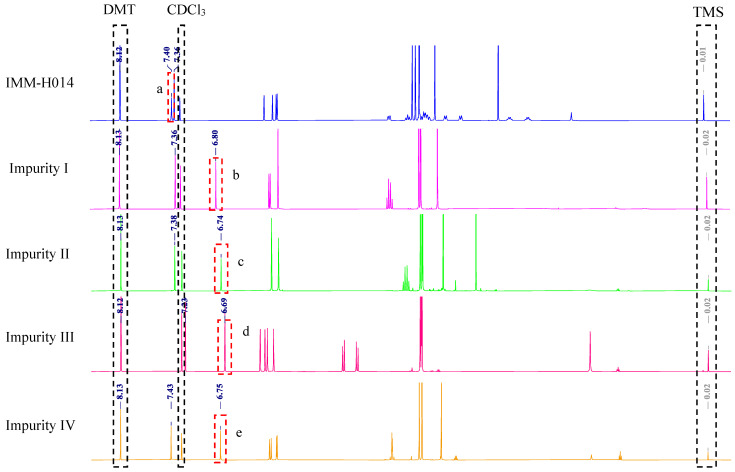
^1^H-NMR spectra of IMM-H014 and its related substances with DMT added in every sample (The red dashed boxes indicate quantitative signals of IMM-H014 and its related substances: a. ^1^H-NMR spectra of IMM-H014 and the expansion of the region *δ* 8.2–7.2 ppm; b. comparison of NMR spectra of IMM-H014 and its related substances in the region of *δ* 8.5~5.0 ppm). a. IMM-H014 H-6′(1H, *δ*7.40, s). b. Impurity I H-6′ (1H, *δ* 6.80, s). c. Impurity II H-6′ (1H, *δ* 6.74, s). d. Impurity III H-6′ (1H, *δ* 6.69, s). e. Impurity IV H-6′ (1H, *δ* 6.75, s). The black dashed boxes indicate signals of DMT, solvent, and TMS.

Moreover, the signal occurs downfield with good separation. Thus, these signals were selected as the quantitative signal. In this study, DMT was used as the IS because the characteristic signal of DMT at *δ* 8.13 ppm (4H, *s*) was well separated from the analyte signals. Therefore, quantification was performed using the signals at 7.40, 6.80, 6.74, 6.69, 6.75, and 8.13 ppm for IMM-H014, impurities I‒IV, and DMT (IS), respectively. Figure 3 shows the ^1^H-NMR spectra of each analyte and DMT.

### 2.3. Selection of Deuterated Solvent

Several deuterated solvents were considered and selected for this experiment, such as deuterated acetone, deuterated dimethyl sulfoxide, and deuterated acetonitrile (ACN). DMT could be dissolved in deuterated dimethyl sulfoxide, ACN, or chloroform, whereas IMM-H014 and impurity II could not be dissolved in dimethyl sulfoxide, and the solubility in ACN was also low. Deuterated chloroform can sufficiently dissolve the individual analytes and IS to meet the quantitative concentration requirements. Moreover, deuterated chloroform is less expensive than other solvents, and its NMR signals (*δ* 7.28 ppm) do not overlap with those of the analytes or the IS. Therefore, the NMR experiments used deuterated chloroform as the ideal solvent.

### 2.4. Optimization of Instrument Parameters

For more accurate quantification and higher sensitivity, we optimized some basic parameters, including pulse angle, relaxation delay (D1), acquisition time (AQ), and time domain (TD) [28]. First, the “Ernat angle” pulse can be calculated using the following formula:

(1) $$cos\alpha =e^{-\frac{T_{R}}{T_{1}}},$$

where α, T_R_, and T_1_ refer to the pulse angle, repetition time, and relaxation time, respectively [29]. Equation (1) shows that the Ernat angle depends on T_1_ for different analytes, while D1, as a part of T_R_, depends on the most extended T_1_ value in the sample. In general, D1 should be five times more than T_1_ to measure 99% of the equilibrium magnetization when 90° is chosen as the angle [30]. However, when 30° is chosen, D1 could be 3/7 times more than T_1_, significantly saving experimental time. Although a 90° pulse theoretically shows better sensitivity, 30° was chosen for this study, which satisfied the quantitative requirements.

The T_1_ values of the quantitative signals were determined for DMT and each analyte (shown in the Table 2 and Figure S6 in Supplementary Materials). In comparison, the T_1_ of DMT H-a was 3.26 s higher than those of other quantitative signals. As shown in Table S1, the peak area of DMT increases with increasing D1 and remains constant as D1 increases to 16 s and beyond. Based on this effect, D1 was set to 20 s as intended for equilibrium magnetization.

AQ and TD are also crucial for obtaining accurate results. An appropriate AQ should avoid interfering with free induction decay (FID), otherwise, “wiggles” will appear in the final spectrum, leading to inaccurate measurements [31]. The TD also affects the qNMR analysis. To obtain a better resolution, we set the AQ value to 6.22 s in this study, and 64 k data points were used for quantitation.

### 2.5. Method Validation

Methodological validation was performed for ^1^H-qNMR, and Table 3, Table 4, Table 5 and Table 6 show the detailed results. 

#### 2.5.1. Specificity

Table 1 lists the chemical shifts of DMT (IS), IMM-H014, and impurities I–IV, and Figure 2 shows the ^1^H-NMR spectra of the IS and all analytes. The figure shows that the baseline is straight and flat above 6 ppm. In addition, the H-6′ signal of each analyte selected for integration did not overlap with other signals from the same molecule, solvent, or IS.

#### 2.5.2. Limit of Quantification (LOQ)

According to Malz and Lancke, the signal-to-noise (*S/N*) ratio must exceed 150:1 to obtain accurate quantitative results with a less than 1% uncertainty. Samples of IMM-H014 and each impurity at minimum concentrations were used to determine the *S/N* ratio and evaluate the limit of quantification (LOQ). The lowest *S/N* ratios of the analytes were 4735.64, 11,763.91, 7472.38, 391.54, and 14,545.91, which indicated the high sensitivity of qNMR.

#### 2.5.3. Robustness

Because IMM-H014 and the related substances are structurally similar, and the same instrument parameters were adopted, IMM-H014 was used as a representative compound to validate robustness. Four crucial instrument parameters were investigated stepwise in wide ranges to assess the robustness of the proposed method. Specifically, pulse length (P1) values between 7.9 and 8.1 affected the results, but the difference calculated using Equation (2) was less than 1%. The number of scans (NS) and TD also did not significantly influence the quantification results. Although D1 substantially affected the results, it exhibited good robustness from 10 to 30 s. Table 3 shows the above results.

(2) $$Diff\%=\frac{\left|P_{x}-99.7\%\right|}{99.7\%}\times 100\%$$

#### 2.5.4. Linearity and Range

Linearity was tested by preparing standard solutions at seven concentrations for each analyte and adding DMT in different molar ratios. The linearity curves were plotted, and the correlation coefficients (*r*) obtained from the calibration curve construction were 0.9999, 0.9999, 0.9999, 0.9997, and 0.9998 for IMM-H014 and its impurities I–IV, respectively. Table 4 indicates that the constructed analytical curves presented a satisfactory linearity for each analyte. The linearity calibration curves of each analyte are shown in Figure S7 in Supplementary Materials. 

#### 2.5.5. Precision and Stability

The precision of the method was estimated by performing six replicate measurements of the mixed standard solution containing the analyte and IS, in which the mass ratio of analytes to IS was approximately eight. As shown in Table 5, the RSD% values were 0.23, 0.44, 1.15, 0.57, and 0.33% for IMM-H014 and its impurities I–IV, respectively. On the other hand, reproducibility was evaluated by parallel analysis of six replicate solutions with the same concentrations of the prepared analyte and IS. The RSD% values were below 1.0%. Solution stability was observed at different time points over 48 h, and the RSD% values were in the range of 0.20–1.02%, indicating good stability at 25 °C. 

#### 2.5.6. Accuracy

In this study, accuracy was characterized using a standard recovery test. Three different known amounts of analyte (at 80, 100, and 120%) were separately added to the system, and then the measured and added amounts were compared. Three parallel samples were prepared in triplicate for each concentration and each analyte. The average recoveries for each analyte were from 98.54 to 100.86%, as shown in Table 6, demonstrating the excellent accuracy of the qNMR method. 

Tables S2–S6 in the Supporting Material present more detailed precision, stability, and accuracy data.

### 2.6. Quantitative Results

The established and validated qNMR method was used to calibrate the APIs of IMM-H014 and its related substances (impurities I–IV), with RSD% values less than 1%, as shown in Table 7. In addition, the same analytes were also calibrated using the mass balance method, where the HPLC% values were obtained by area normalization, and the loss on drying% values were determined via the thermogravimetric (TG) method (show in Figure S8 in the Supplementary Material for details). No considerable differences were found between the quantitative results using the proposed qNMR method and the conventional mass balance method for all samples. However, qNMR does not require the determination of the loss on drying% value, which can simplify the determination process and save samples. Thus, the qNMR method is more suitable for the purity calibration of samples with high prices and insufficient dosage, but it is not suitable for quantitative analysis of compounds with severely occluded and overlapped spectral peaks since the display space of ^1^H spectrum is only about 20 ppm.

## 3. Materials and Methods

### 3.1. Materials

IMM-H014 (batch No. 20211001) and impurities I, II, III, and IV (batch Nos. 20190301, 20190418, 20180911, and 20191204, respectively) were synthesized by our laboratory, and their structures were identified spectroscopically (spectral data are shown in Supplementary Material Figures S1–S5). The DMT reference standard (purity 100%, internal standard for qNMR) was purchased from the Chinese National Institutes for Food and Drug Control (NIFDC, Beijing, China, Cat. No. 510052-201401). Deuterated chloroform (purity 99.8%) containing 0.03% TMS and silver foil as the stabilizer was bought from Adamas Pharmaceuticals, Inc. (Shanghai, China). Acetonitrile, triethylamine, and HPLC grade methanol (MeOH) were purchased from innoChem Science and Technology Co., Ltd. (Beijing, China). The distilled water used was purified water bought from Hangzhou Wahaha Group Co., Ltd., Hangzhou, China.

### 3.2. Instrument

HPLC analysis was performed on a Thermo Ultimate U3000 liquid chromatograph equipped with a DAD detector (Thermo Fisher Scientific Co., Ltd., Waltham, MA, USA). An ODS C18 column (4.6 mm × 250 mm, particle size of 5 μm) maintained at 35 °C was used for preparation. TG analysis was performed on a METTLER TOLEDO TGA/DSC-1 (Mettler Toledo International Co., Ltd., Hongkong, China).

All samples were weighed using one over a million on an electronic balance (XP-6, Sartorius). All spectra were processed using Bruker’s Topspin software (version 3.6.2, Bruker Biospin, Spring, TX, USA). All chemical shifts are reported in ppm relative to TMS at 0.00 ppm. qNMR experiments were performed with the following optimized parameters: pulse angle, 30; pulse width, 2.4 μs; data points, 64 K; NS, 32; D1, 20 s; TD, 64 K; P1, 8 μs; AQ, 6.22 s; and spectral width (SW), 13.0174 ppm. A line-broadening factor of 0.20 Hz was applied to FIDs before Fourier transformation. The repetition delay was 30 s, calculated using the inversion recovery pulse program. T_1_ measuring experiments in this study were performed with the following parameters: pulse program, t1ir; NS, 8; D1, 20 s; TD, 16 K; and P1, 8 μs. The T_1_ relaxation times for the target protons are as follows: for IMM-H014, *δ* 7.42 ppm (H-6’), T_1_ = 0.48 s; for impurity I, *δ* 6.80 ppm, T_1_ = 1.65 s; for impurity II, *δ* 6.74 ppm, T_1_ = 1.51 s; for impurity III, *δ* 6.69 ppm, T_1_ = 1.20 s; for impurity IV, *δ* 6.75 ppm, T_1_ = 1.61 s; and for DMT, *δ* 8.13 ppm, T_1_ = 3.26 s. All data were managed using MestReNova v14.0.0-23239.

### 3.3. Sample Preparation

After weighing the internal standard and the analytes of the corresponding mass using the electronic balance, the samples were dissolved into 0.8–1.0 mL of deuterated chloroform and sonicated for 1 min for mixing. We put 0.6 mL into 5 mm NMR tubes to measure the ^1^H-NMR spectra. For T_1_ measurements, the sample preparation procedure was essentially the same as described above, except that no internal standard was added.

In the HPLC method, IMM-H014 and its impurities I‒IV were prepared at 0.5 mg/mL using a solvent of a mixture of water and ACN in the proportion of 70:30 (*v*/*v*), respectively.

### 3.4. qNMR Analysis Method

All samples were determined three times, and the average value was calculated. Formula (3) [32] was used to estimate the absolute purity *Px*, and the results are shown in Table 6.

(3) $$P_{x}=P_{std}\times \frac{N_{std}}{N_{x}}\frac{M_{x}}{M_{std}}\frac{A_{x}}{A_{std}}\frac{m_{std}}{m_{x}}\times 100\%$$

where *P_std_* is the purity of the IS (DMT, purity 100%); *N_std_* and *Nx* refer to the number of quantitative protons of the IS and analytes, respectively; *M_x_* and *M_std_* refer to the molecular weights of the IS and analytes, respectively; *A_x_* is the integral value of the peak of the IS quantitative signal, while *A_std_* is the integral value of the area of the quantitative signal of the analyte. Then, *m_std_* and *m_x_* correspond to the weights of the IS and analytes, respectively. Table 8 provides specific information about IS, IMM-H014, and its impurities.

### 3.5. Method Validation

The abovementioned method was validated using seven parameters: specificity, robustness, linearity, range, precision, accuracy, and solution stability. In the specificity test, the weight of IMM-H014 and its impurities should be at least eight times that of IS to integrate the quantitative signal similarly. IMM-H014 was selected as a typical example in terms of robustness. Seven solutions with concentrations from low to high were prepared and tested for every analyte. Furthermore, the correlation coefficient (*r*) and slope were calculated using the least squares method. Table 4 shows the results. Precision, reproducibility, and repeatability were assessed using six different solutions and six replicated measurements of the same solution. The results were evaluated by calculating RSD%, and Table 5 provides detailed information. The accuracy of this method was characterized by the recovery test, in which the accurate amounts of the analytes (at 80, 100, and 120%) were added to the exact quantity and the experimental and theoretical values. Solution stability was determined by assessing the solution at different points (0, 1, 2, 4, 8, 12, 24, 36, and 48 h). 

### 3.6. HPLC Method

The HPLC method was established and validated previously [18] and was used to quantify the analytes in this study. HPLC was performed on a Thermo Ultimate U3000 LC with a DAD detector (Thermo Fisher Scientific Co., Ltd.). An ODS C18 column (4.6 mm × 250 mm, 5 μm) was used for separating IMM-H014 and its impurities at 35 °C. Mobile phase A was CAN, and mobile phase B was 0.1% (*v*/*v*) formic acid (pH adjusted to 4.0 with triethylamine) mixed with ACN in a constant proportion of 70:30 (*v*/*v*). Gradient elution was used at a 0.7 mL/min flow speed as follows: The percentage of B was 100% at 0–15 min and was decreased from 100 to 70% at 15–30 min. It was then held at 70% at 30‒40 min. The DAD wavelength was set at 230 nm. 

### 3.7. TG Method

TG analysis was performed on a TGA/DSC-1. Approximately 7 mg of the analytes was used for this measurement. We added the sample to a 70 μL alumina crucible and analyzed it with the TGA. The setting conditions included a heating rate of 10 k/min and a heating interval of 40–400 °C. Moreover, the gas in the furnace was nitrogen, and the flow rate was 20 mL/min.

## 4. Conclusions

In this study, a new rapid, simple, sensitive, and selective qNMR method was established and applied to the purity assessment of IMM-H014, a new drug for treating MAFLD and its critical related substances (impurities I–IV). DMT was used as the IS, and deuterated chloroform was used as the solvent. The aromatic protons (6′-H) from the skeleton of the analytes were selected as the quantitative signals. Several key experimental parameters were explored and optimized, including D1, AQ, and TD. Regarding the methodology, verifying specificity, robustness, linearity, LOQ, precision, stability, and accuracy indicated that the qNMR method was a reliable and practical tool for purity assessment. In addition, the results were consistent with those from the traditional mass balance method.

Compared with the mass balance method, the qNMR method proposed in this study does not require the determination of the loss on drying rate, which simplifies the determination process, saves samples, and is more suitable for the purity calibration of expensive and insufficient amounts of samples. Therefore, the qNMR method has obvious advantages which could ensure the reliability of the RMs required in subsequent quality control studies and help ensure the safe use of new drugs during clinical treatments.

## Figures and Tables

**Table 1:** The assignments of ^1^H-NMR chemical shifts for IMM-H014 and its related substances.

Compound	IMM-H014	Impurity I	Impurity II	Impurity III	Impurity IV
H-2	2H 6.05 dd	2H 6.06 dd	2H 6.05 s	1H 6.20 d	2H 6.06 dd
				1H 6.10 d	
H-2′	2H 5.94 dd	2H 5.94 dd	2H 5.95 dd	1H 6.14 d	2H 5.97 dd
				1H 6.02 d	
H-6	1H 7.36 s	1H 7.36 s	1H 7.38 s	1H 7.23 s	1H 7.43 s
**H**-6′ *	**1H 7.40 s**	**1H 6.80 s**	**1H 6.74 s**	**1H 6.69 s**	**1H 6.75 s**
H-8	-	2H 4.40 q	2H 4.22 dd	1H 5.05 s	2H 4.38 d
				1H 4.86 s	
H-9	3H 3.71 s	3H 3.74 s	3H 3.68 s	-	3H 3.70 s
H-9′	-	-	3H 3.22 s	-	-
H-15	3H 4.02 s	8H 3.98 m	8H 3.97 m	8H 3.98 m	3H 4.01 s
H-15′	3H 3.98 s	8H 3.98 m	8H 3.97 m	8H 3.98 m	5H 3.97 m
H-8′	1H 4.33 dd	8H 3.98 m	8H 3.97 m	8H 3.98 m	5H 3.97 m
	1H 3.79 dd				

* Bold values represent quantitative signals and their chemical shifts.

**Table 2:** T_1_ and chemical shift of quantitative signal.

Compound	Chemical Shift/ppm	T_1_/s
IMM-H014	7.42	0.48
Impurity I	6.80	1.65
Impurity II	6.74	1.51
Impurity III	6.69	1.20
Impurity IV	6.75	1.61
DMT	8.13	3.26

**Table 3:** Robustness of the proposed method.

Parameters	Value	Px/%	Diff%
Relaxation Delay (D1)	10 s	99.76%	0.002
	15 s	99.72%	0.045
	**20 s ^#^**	99.76%	/
	25 s	100.29%	0.533%
	30 s	99.76%	0.002%
Number of Scans (NS)	16	99.93%	0.169%
	**32 ^#^**	99.76%	/
	64	99.22%	0.537%
Pulse Length (P1)	7.90 μs	99.61%	0.151%
	7.95 μs	99.88%	0.117%
	**8.00 μs ^#^**	99.76%	**/**
	8.05 μs	99.61%	0.151%
	8.10 μs	98.79%	0.968%
Time Domain (TD)	32 k	99.66%	0.105%
	**64 k ^#^**	99.76%	**/**

**^#^** Bold values represent standard parameter sets.

**Table 4:** Linearity and range test of IMM-H014 and its related substances by qNMR.

No.	IMM-H014		Impurity I		Impurity II		Impurity III		Impurity IV	
	*m_x_/m_std_*	*A_x_/A_std_*	*m_x_/m_std_*	*A_x_/A_std_*	*m_x_/m_std_*	*A_x_/A_std_*	*m_x_/m_std_*	*A_x_/A_std_*	*m_x_/m_std_*	*A_x_/A_std_*
1	0.4247	0.0360	0.4999	0.0625	0.5074	0.0607	0.4754	0.0637	0.5538	0.0650
2	1.0336	0.0901	0.9601	0.1201	0.8251	0.0987	1.5862	0.2134	1.1027	0.1286
3	2.1934	0.1914	2.0247	0.2519	1.7319	0.2076	1.9346	0.2607	1.6546	0.1944
4	2.9762	0.2598	3.3830	0.4241	3.0961	0.3719	3.2555	0.4379	3.7443	0.4436
5	7.3463	0.6399	6.5211	0.8005	5.0897	0.6146	5.6846	0.7644	5.9357	0.6965
6	14.6494	1.2693	16.0871	1.9684	12.4175	1.4975	14.0762	1.8927	15.1153	1.8020
7	31.7979	2.7083	21.5391	2.7018	31.2684	3.6506	18.3831	2.5560	25.8372	2.9900
Calibration	y = 0.0852x + 0.0066		y = 0.1244x − 0.0028		y = 0.1168x + 0.012		y = 0.1379x − 0.0102		y = 0.1163x + 0.0067	
*r*	0.9999		0.9999		0.9999		0.9997		0.9998	

**Table 5:** Precision, reproducibility, and stability of IMM-H014 and its related substances by qNMR.

		IMM-H014			Impurity I			Impurity II			Impurity III			Impurity IV		
No.		*m_x_*/ *m_std_*	*A_x_*/ *A_std_*	%	*m_x_*/ *m_std_*	*A_x_*/ *A_std_*	%	*m_x_*/ *m_std_*	*A_x_*/ *A_std_*	%	*m_x_*/ *m_std_*	*A_x_*/ *A_std_*	%	*m_x_*/ *m_std_*	*A_x_*/ *A_std_*	%
Precision (*n* = 6)	1	7.346	0.637	99.27	6.521	0.805	99.20	5.090	0.613	100.27	5.677	0.769	100.57	5.936	0.694	98.49
	2		0.641	99.80		0.797	98.27		0.618	101.22		0.767	99.70		0.699	99.12
	3		0.639	99.61		0.797	98.27		0.613	100.27		0.765	99.68		0.697	98.81
	4		0.639	99.61		0.799	98.46		0.607	99.33		0.767	99.88		0.694	98.49
	5		0.637	99.27		0.799	98.46		0.601	98.38		0.763	100.42		0.697	98.90
	6		0.640	99.76		0.805	99.20		0.601	98.38		0.759	98.98		0.692	98.21
Average		/	/	99.55	/	/	98.64	/	/	99.64	/	/	99.87	/	/	98.67
RSD%		/	/	0.23	/	/	0.44	/	/	1.15	/	/	0.57	/	/	0.33
Repeatability (*n* = 6)	1	7.346	0.640	99.67	6.521	0.800	98.58	5.090	0.615	100.59	5.685	0.769	99.57	5.936	0.697	98.81
	2	5.851	0.511	99.90	4.846	0.599	99.33	4.846	0.579	99.49	3.809	0.512	99.13	7.768	0.919	99.58
	3	7.957	0.694	99.85	3.884	0.480	99.46	7.310	0.894	101.82	5.168	0.695	99.15	8.395	0.990	99.27
	4	5.889	0.513	99.60	7.772	0.965	99.86	7.772	0.929	99.52	3.809	0.513	99.30	5.064	0.601	99.97
	5	8.091	0.707	100.00	6.736	0.841	100.35	6.736	0.806	99.58	5.241	0.703	99.56	7.607	0.894	98.95
	6	4.884	0.426	99.82	6.030	0.751	100.17	6.03	0.728	100.51	4.399	0.591	99.31	8.242	0.969	98.99
Average		/	/	99.81	/	/	99.63	/	/	100.25	/	/	99.34	/	/	99.26
RSD%		/	/	0.38	/	/	0.70	/	/	0.91	/	/	0.33	/	/	0.67
Stability	0 *	7.346	0.637	99.27	6.521	0.805	99.20	5.090	0.613	100.27	5.677	0.769	99.93	5.936	0.694	98.49
	1		0.641	99.80		0.797	98.27		0.618	101.22		0.767	99.70		0.699	99.12
	2		0.639	99.61		0.797	98.27		0.613	100.27		0.765	99.48		0.697	98.81
	4		0.639	99.61		0.799	98.46		0.607	99.33		0.767	99.70		0.694	98.49
	8		0.637	99.27		0.799	98.46		0.601	98.38		0.763	99.19		0.697	98.90
	12		0.640	99.76		0.805	99.20		0.601	98.38		0.759	98.73		0.692	98.21
	24		0.641	99.80		0.791	97.54		0.601	98.38		0.766	99.64		0.7	99.25
	36		0.639	99.61		0.797	98.27		0.606	99.13		0.764	99.26		0.702	99.60
	48		0.639	99.61		0.805	99.20		0.605	98.95		0.763	99.19		0.702	99.59
Average		/	/	99.59	/	/	98.54	/	/	99.37	/	/	99.43	/	/	98.94
RSD%		/	/	0.20	/	/	0.57	/	/	1.02	/	/	0.37	/	/	0.50

* Time in h.

**Table 6:** Recovery test of IMM-H014 and its related substances by qNMR.

		IMM-H014			Impurity Ⅰ			Impurity Ⅱ			Impurity Ⅲ			Impurity Ⅳ		
	No.	*m_x_*/ *m_std_*	*A_x_*/ *A_std_*	*%*	*m_x_*/ *m_std_*	*A_x_*/ *A_std_*	*%*	*m_x_*/ *m_std_*	*A_x_*/ *A_std_*	*%*	*m_x_*/ *m_std_*	*A_x_*/ *A_std_*	*%*	*m_x_*/ *m_std_*	*A_x_*/ *A_std_*	*%*
Accuracy at low level (*n* = 3)																
	1	4.884	0.426	99.82	5.300	0.657	99.62	5.090	0.615	100.59	3.804	0.513	99.44	4.279	0.507	99.79
	2		0.426	99.82		0.657	99.71		0.603	98.70		0.512	99.33		0.506	99.54
	3		0.426	99.82		0.657	99.69		0.604	98.82		0.511	99.24		0.503	98.97
Average		/	/	99.82	/	/	99.67	/	/	99.37	/	/	99.34	/	/	99.43
RSD%		/	/	0.61	/	/	0.59	/	/	1.02	/	/	0.20	/	/	0.56
Accuracy at medium level (*n* = 3)																
	1	5.889	0.511	99.90	6.521	0.8	98.58	5.878	0.699	99.07	5.677	0.767	99.70	5.936	0.697	98.81
	2		0.513	99.60		0.801	98.71		0.702	99.45		0.763	99.21		0.694	98.53
	3		0.426	99.82		0.798	98.34		0.702	99.48		0.764	99.36		0.701	99.48
Average		/	/	99.81	/	/	98.54	/	/	99.33	/	/	99.43	/	/	98.94
RSD%		/	/	0.52	/	/	0.57	/	/	0.38	/	/	0.37	/	/	0.50
Accuracy at high level (*n* = 3)																
	1	7.957	0.640	99.67	7.895	0.987	100.53	8.046	0.958	99.14	7.508	1.017	100.01	9.842	1.160	99.31
	2		0.694	99.85		0.990	100.86		0.961	99.44		1.016	99.85		1.154	98.70
	3		0.707	100.00		0.990	100.86		0.963	99.68		1.014	99.65		1.164	99.54
Average		/	/	99.81	/	/	100.75	/	/	99.42	/	/	99.84	/	/	99.18
RSD%		/	/	0.19	/	/	0.33	/	/	0.92	/	/	0.53	/	/	0.80

**Table 7:** Comparison of purity determination results between qNMR method and mass balance method.

Compound	qNMR Method (*n* = 3)		Mass Balance Method		
	Purity (%)	RSD (%)	HPLC (%)	Loss on Drying (%)	Purity (%) *
IMM-H014	99.81	0.46	99.52	0.022	99.50
Impurity I	99.65	1.04	99.64	0.085	99.91
Impurity II	99.37	0.79	99.18	0.032	99.15
Impurity III	99.29	0.27	99.58	0.098	99.48
Impurity IV	99.19	0.64	99.50	0.936	99.06

* Purity% = HPLC% × (100% − loss on drying%).

**Table 8:** Specific information about the quantitative signal of the compounds.

Name	N	M (g/mol)
DMT(IS)	4	194.18
IMM-H014	1	555.55
Impurity I	1	390.34
Impurity II	1	404.37
Impurity III	1	358.30
Impurity IV	1	408.79

## Data Availability

Data is contained within the article and supplementary material.

## Supplementary Materials

The following supporting information can be downloaded at: https://www.mdpi.com/article/10.3390/ijms242417508/s1.
