# Supplementary material for: Assessment of the Purity of IMM-H014 and Its Related Substances for the Treatment of Metabolic-Associated Fatty Liver Disease Using Quantitative Nuclear Magnetic Resonance Spectroscopy
Source: Int J Mol Sci. 2023 Dec 15;24(24):17508. doi: 10.3390/ijms242417508 (PMC10744271; doi:10.3390/ijms242417508)
Supplement: Supplementary file 1 [file ijms-24-17508-s001.zip › ijms-2754646-supplementary.pdf]

Supplementary Materials

Assessing the Purity of IMM-H014 and Its Related Substances for the Treatment of Non-Alcoholic Fatty Liver Disease Using Quantitative Nuclear Magnetic Resonance Method

Hanyilan Zhang†,[1] Haowen Zhu†,[1] Xiaoliang Gong,[1] Haoyang Tang,[1] Wenxuan Zhang,[1] Tiesong Wang,[2] Song Wu,[1] Yinghong Wang*,[1] Qingyun Yang*[1]

1 State Key Laboratory of Bioactive Substance and Function of Natural Medicines, Institute of Materia Medica, Chinese Academy of Medical Sciences & Peking Union Medical College, Beijing 100050, China

2. NMPA Key Laboratory for Research and Evaluation of Generic Drugs, Beijing Institute for Drug Control, Beijing 102206, China

Correspondence*: yqy@imm.ac.cn; TEL:13683270346

Contents

Figure S1. 1H NMR, 13C NMR, and HR-ESI–MS spectral data of IMM-H014 ... 1-2

Figure S2. 1H NMR, 13C NMR, and HR-ESI–MS spectral data of impurity I ... 3-4

Figure S3. 1H NMR, 13C NMR, and HR-ESI–MS spectral data of impurity II ... 5-6

Figure S4. 1H NMR, 13C NMR, and HR-ESI–MS spectral data of impurity III ... 7-8

Figure S5. 1H NMR, 13C NMR, and HR-ESI–MS spectral data of impurity IV ... 9-10

Figure S6. T1 relaxation time of quantitative signal of DMT, IMM-H014 and impurities I–IV ... 11-13

Figure S7. Linearity calibration curves of IMM-H014 and its related substances ... 14-15

Figure S8. TG test of IMM-H014 and Impurity I-IV ... 16-17

Table S1. Integration using different D1 value ... 18

Table S2. Precision, accuracy and stability test of IMM-H014 ... 18

Table S3. Precision, accuracy and stability test of Impurity I ... 19

Table S4. Precision, accuracy and stability test of Impurity II ... 20

Table S5. Precision, accuracy and stability test of Impurity III ... 21

Table S6. Precision, accuracy and stability test of Impurity IV ... 22

Figure S1. ^{1}H NMR, ^{13}C NMR, and HR-ESI–MS spectral data of IMM-H014

Methyl 7, 7'-dimethoxy-5'-(morpholinomethyl)-[4, 4'-bibenzo[d][1,3]dioxole]-5-carboxylate methanesulfonate

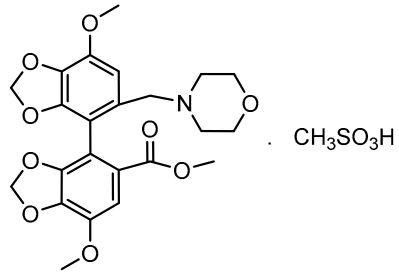

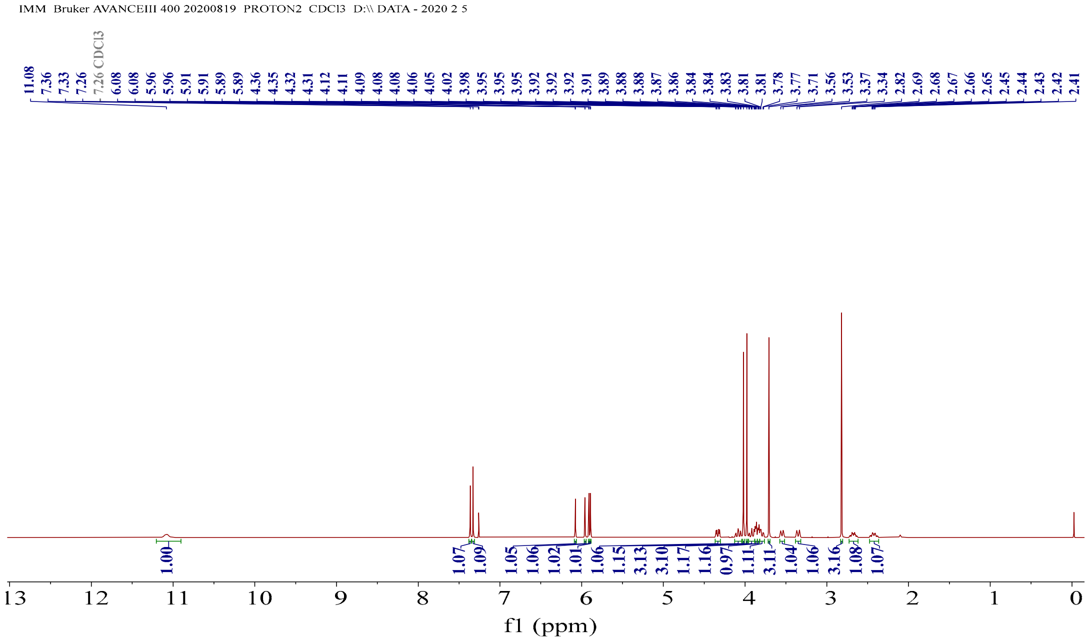

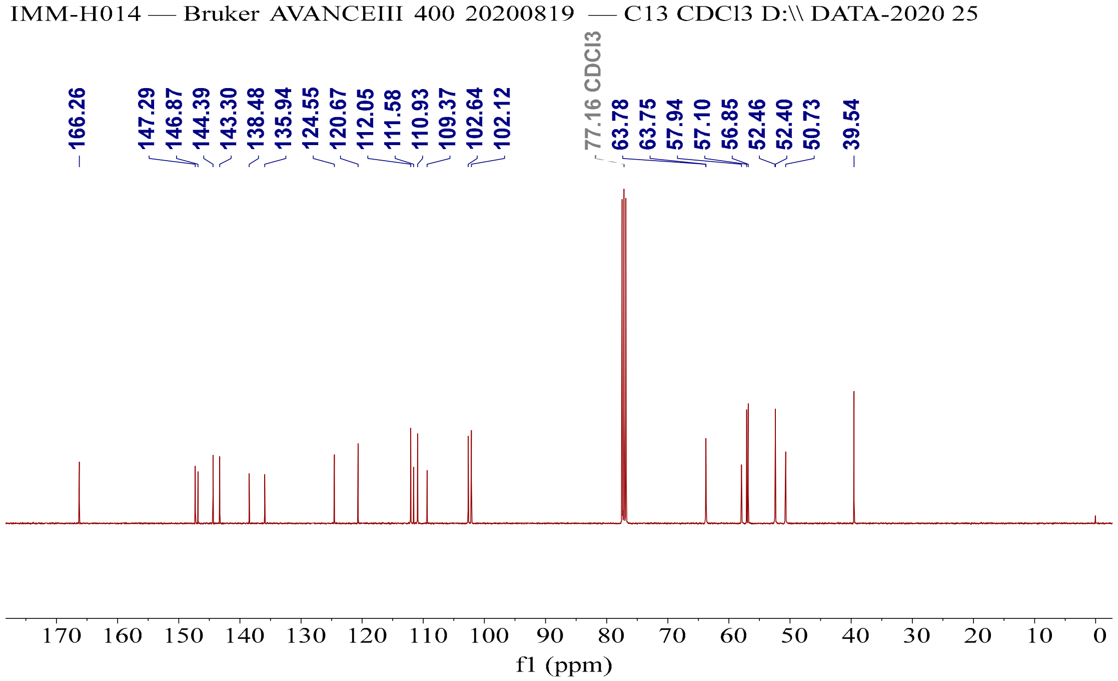

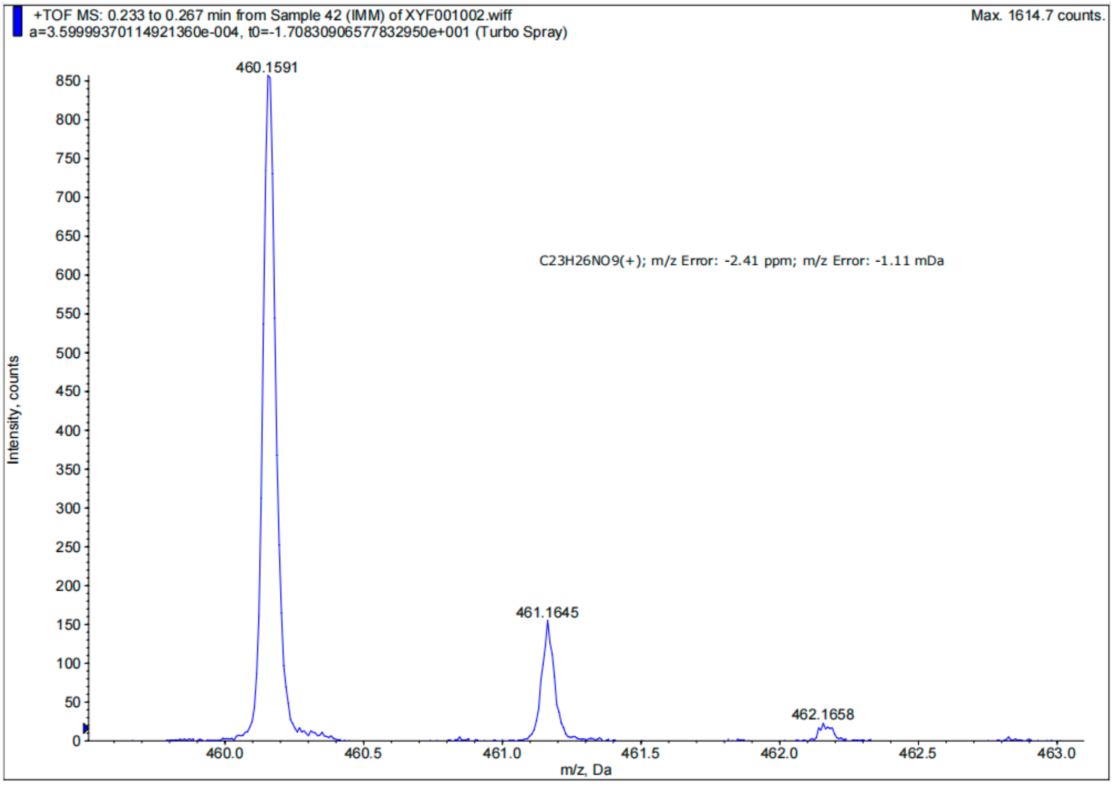

^{1}H NMR (400 MHz, $CDCl_3$) δ 11.08 (s, 1H), 7.36 (s, 1H), 7.33 (s, 1H), 6.08 (d, J = 1.2 Hz, 1H), 5.96 (d, J = 1.2 Hz, 1H), 5.91 (d, J = 1.4 Hz, 1H), 5.89 (d, J = 1.4 Hz, 1H), 4.33 (dd, J = 13.2, 4.2 Hz, 1H), 4.08 (m, J = 13.7, 11.8, 2.2 Hz, 1H), 4.02 (s, 3H), 3.98 (s, 3H), 3.92 (m, J = 12.3, 11.5, 1.8 Hz, 1H), 3.87 (d, J = 6.6 Hz, 1H), 3.85 – 3.82 (m, 1H), 3.79 (dd, J = 13.1, 3.4 Hz, 1H), 3.71 (s, 3H), 3.55 (d, J = 12.1 Hz, 1H), 3.35 (d, J = 12.1 Hz, 1H), 2.82 (s, 3H), 2.67 (m, J = 12.1, 8.8, 3.8 Hz, 1H), 2.48 – 2.36 (m, 1H). ^{13}C NMR (101 MHz, $CDCl_3$) δ 166.26, 147.29, 146.87, 144.39, 143.30, 138.48, 135.94, 124.55, 120.67, 112.05, 111.58, 110.93, 109.37, 102.64, 102.12, 63.78, 63.75, 57.94, 57.10, 56.85, 52.46, 52.40, 50.73, 39.54. HR-ESI–MS: *m/z* [M–CH_3SO_3H+H]$^+$ (organic base) calcd for $C_{23}H_{25}NO_9$ 460.1529, found 460.1591.

Figure S2. ^{1}H NMR, ^{13}C NMR, and HR-ESI–MS spectral data of impurity I

Methyl 5'-(hydroxymethyl)-7,7'-dimethoxy-[4,4'-bibenzo[d][1,3]dioxole]-5-carboxylate

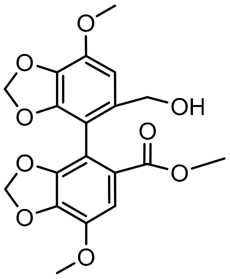

20220424 impurityⅡ-CDCl3.1.1.1r Bruker AVANCEIII 400 20220424
PROTON2 CDCl3 D:\\ DATA-2022 8

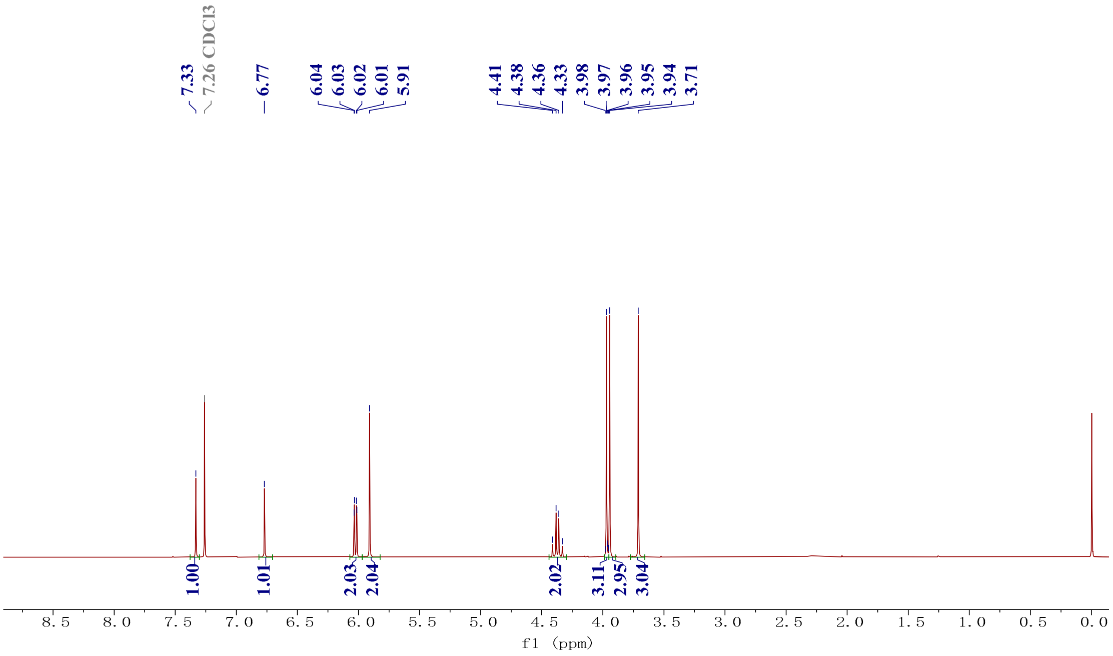

20230530 Impurity II.1.fid — Bruker AVANCE III 400 — C13 CDCl3 D:\\ DATA-2023 15

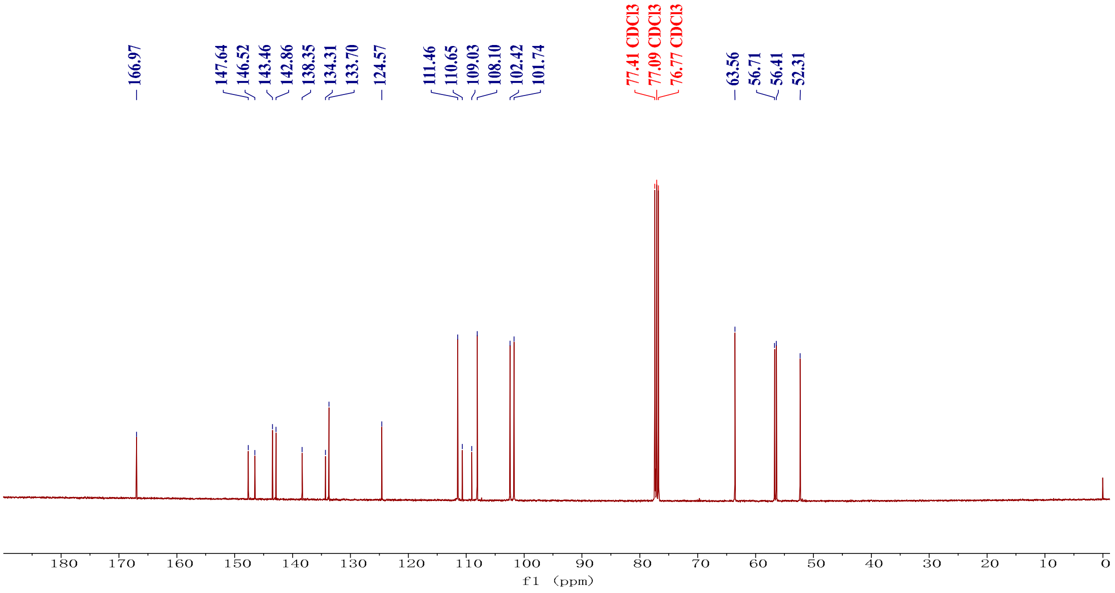

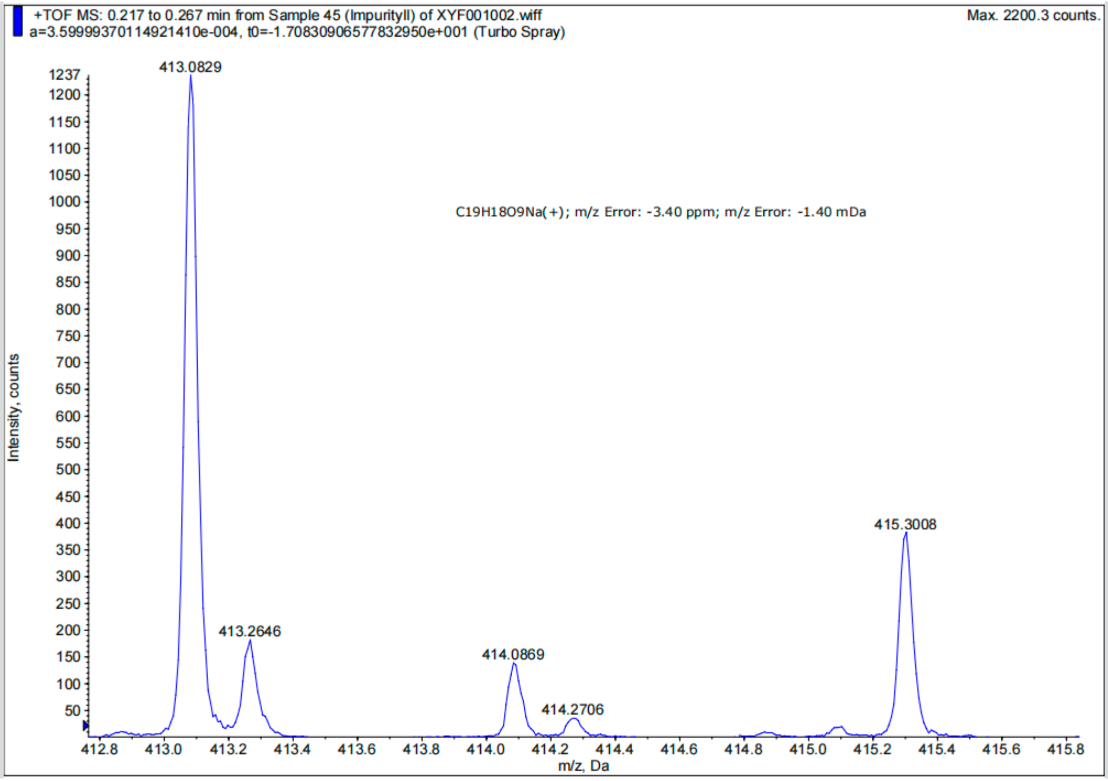

^{1}H NMR (400 MHz, $CDCl_3$) δ 7.33 (s, 1H), 6.77 (s, 1H), 6.03 (q, 2H), 5.91 (s, 2H), 4.37 (q, 2H), 3.97 (s, 3H), 3.94 (s, 3H), 3.71 (s, 3H). ^{13}C NMR (101 MHz, $CDCl_3$) δ 166.97, 147.64, 146.52, 143.46, 142.86, 138.35, 134.31, 133.70, 124.57, 111.46, 110.65, 109.03, 108.10, 102.42, 101.74, 63.56, 56.71, 56.41, 52.31. HR-ESI–MS: *m/z* $[M+Na]^+$ calcd for $C_{19}H_{18}O_9$ 413.0951, found 413.0829.

Figure S3. ^{1}H NMR, ^{13}C NMR, and HR-ESI–MS spectral data of impurity II

Methyl 7,7'-dimethoxy-5'-(methoxymethyl)-[4,4'-bibenzo[d][1,3]dioxole]-5-carboxylate

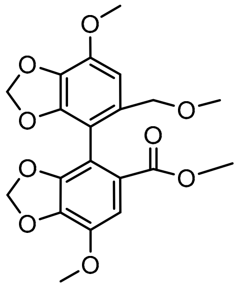

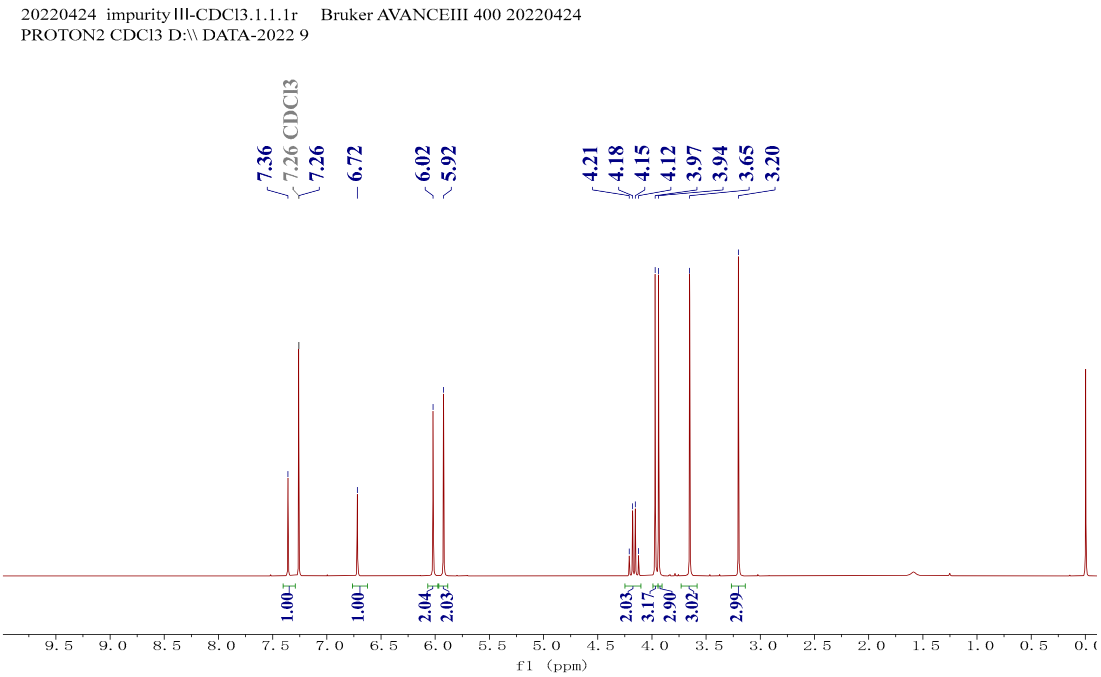

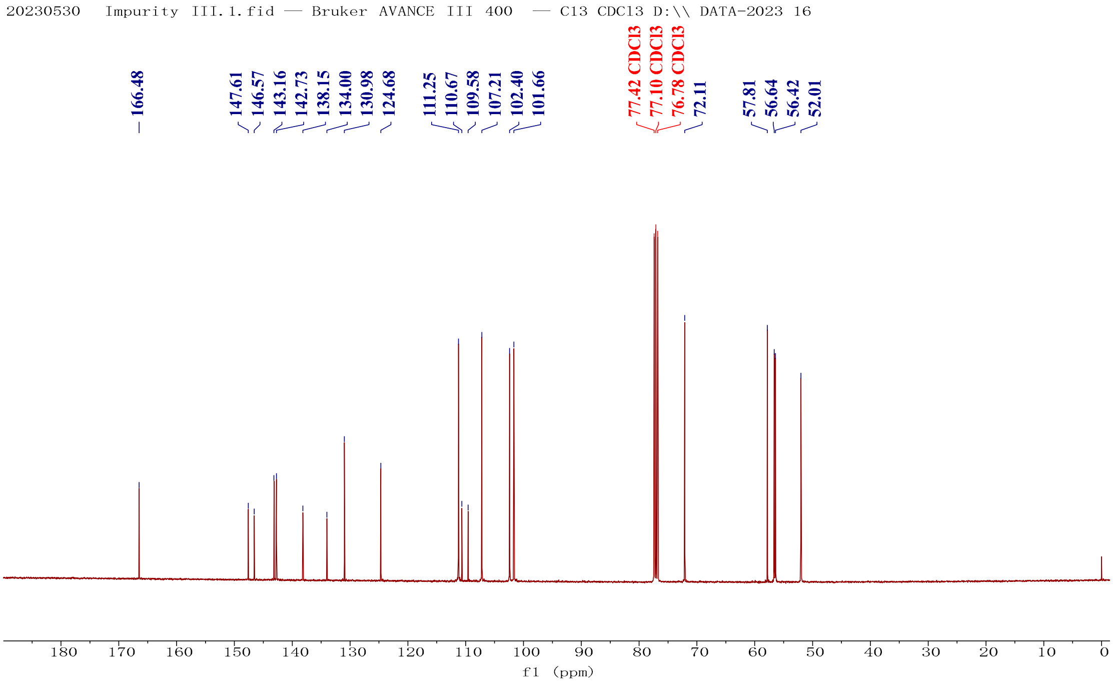

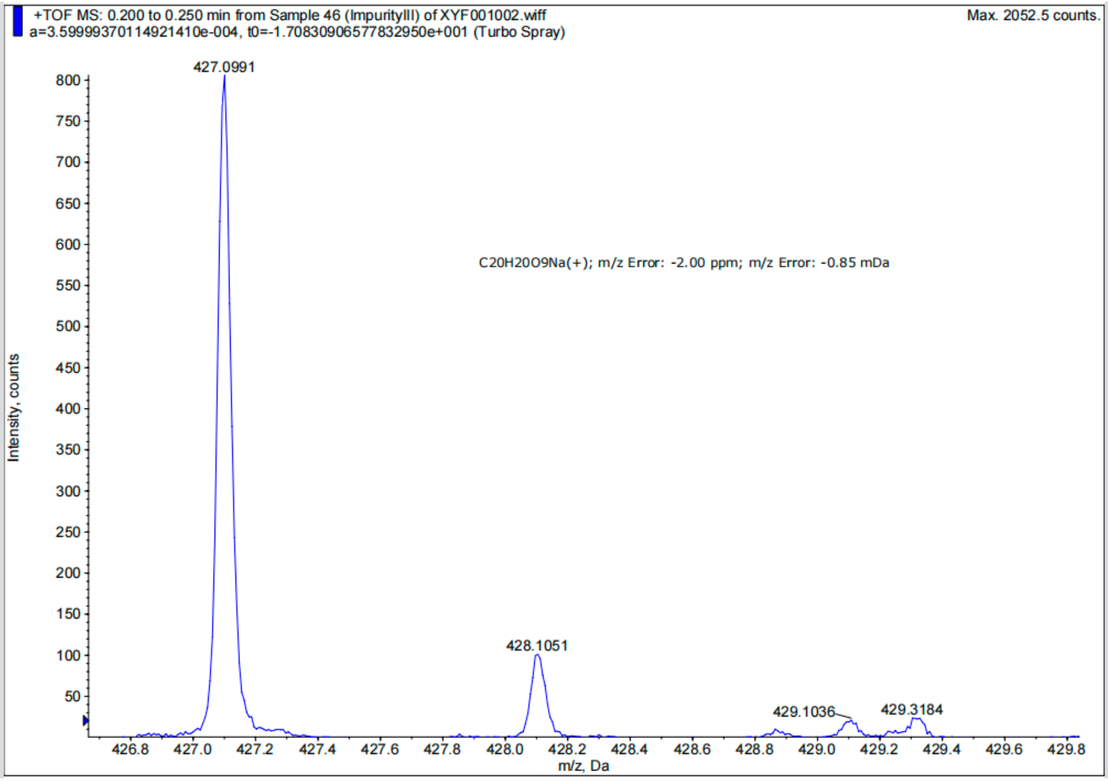

^{1}H NMR (400 MHz, $CDCl_3$) δ 7.36 (s, 1H), 6.72 (s, 1H), 6.02 (s, 2H), 5.92 (s, 2H), 4.16 (q, 2H), 3.97 (s, 3H), 3.94 (s, 3H), 3.65 (s, 3H), 3.20 (s, 3H). ^{13}C NMR (101 MHz, $CDCl_3$) δ 166.48, 147.61, 146.57, 143.16, 142.73, 138.15, 134.00, 130.98, 124.68, 111.25, 110.67, 109.58, 107.21, 102.40, 101.66, 72.11, 57.81, 56.64, 56.42, 52.01. HR-ESI–MS: *m/z* $[M+Na]^+$ calcd for $C_{20}H_{20}O_9$ 427.1107, found 427.0991.

Figure S4. ^{1}H NMR, ^{13}C NMR, and HR-ESI–MS spectral data of impurity Ⅲ

4,10-dimethoxy-[1,3]dioxolo[4',5':3,4]benzo[1,2-*c*][1,3]dioxolo[4',5':5,6]benzo[1,2-*e*]oxepin-6(8H)-one

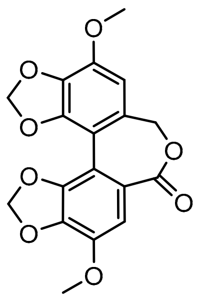

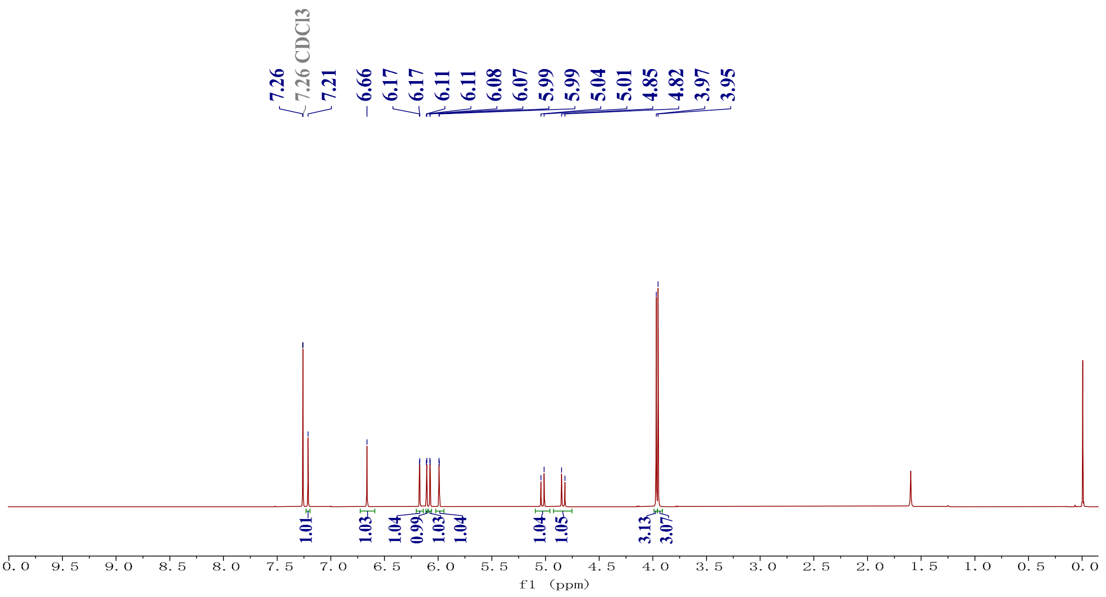

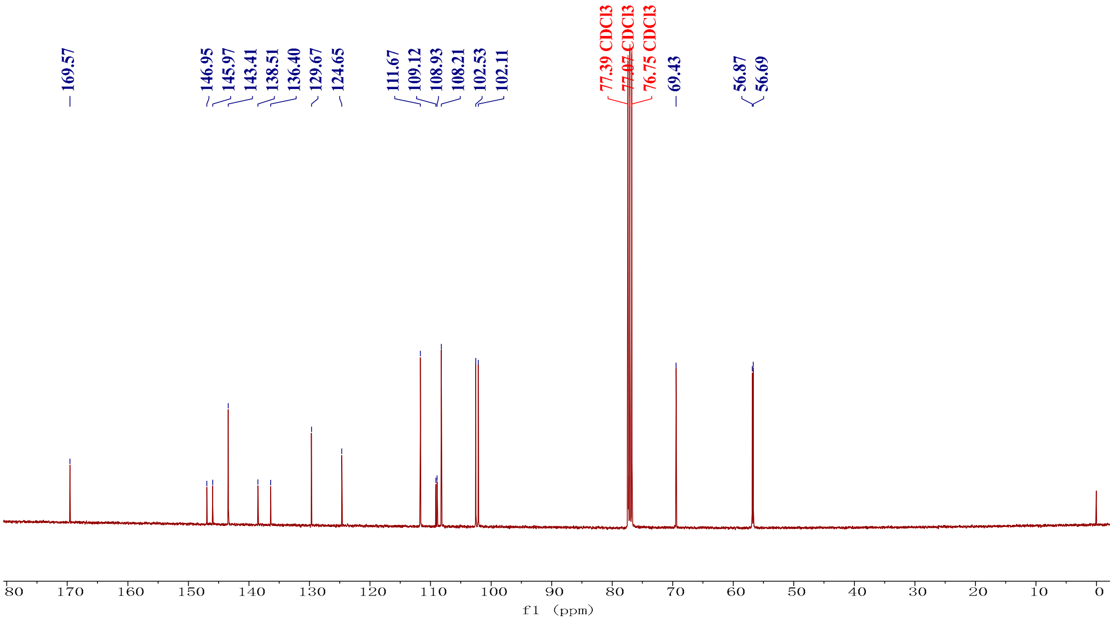

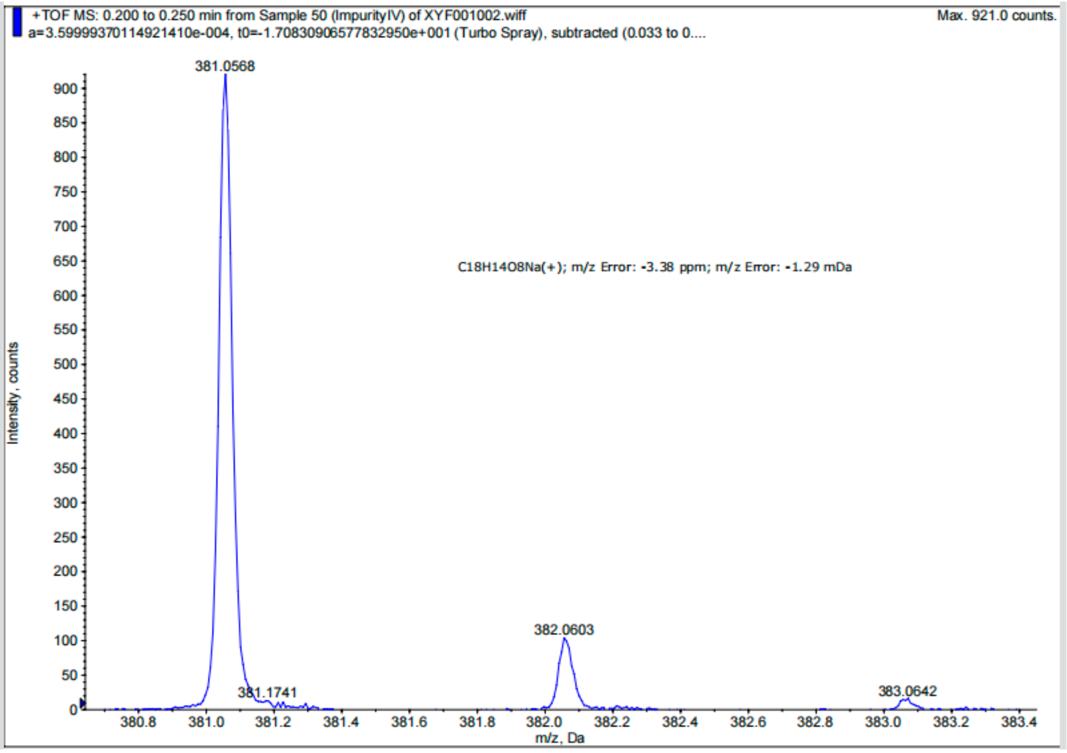

^{1}H NMR (400 MHz, $CDCl_3$) δ 7.21 (s, 1H), 6.67 (s, 1H), 6.17 (d, J = 1.4 Hz, 1H), 6.11 (d, J = 1.5 Hz, 1H), 6.08 (d, J = 1.5 Hz, 1H), 5.99 (d, J = 1.4 Hz, 1H), 5.03 (d, J = 12.2 Hz, 1H), 4.83 (d, J = 12.2 Hz, 1H), 3.97 (s, 3H), 3.95 (s, 3H). ^{13}C NMR (101 MHz, $CDCl_3$) δ 169.57, 146.95, 145.97, 143.41, 138.51, 136.40, 129.67, 124.65, 111.67, 109.12, 108.93, 108.21, 102.53, 102.11, 69.43, 56.87, 56.69. HR-ESI/APCI–MS: *m/z* $[M+H]^+$ calcd for $C_{18}H_{14}O_8$ 381.0689, found 381.0568.

Figure S5. ^{1}H NMR, ^{13}C NMR, and HR-ESI–MS spectral data of Impurity Ⅳ

Methyl 5'-(chloromethyl)-7,7'-dimethoxy-[4,4'-bibenzo[d][1,3]dioxole]-5-carboxylate

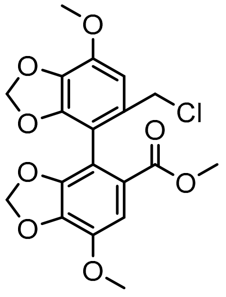

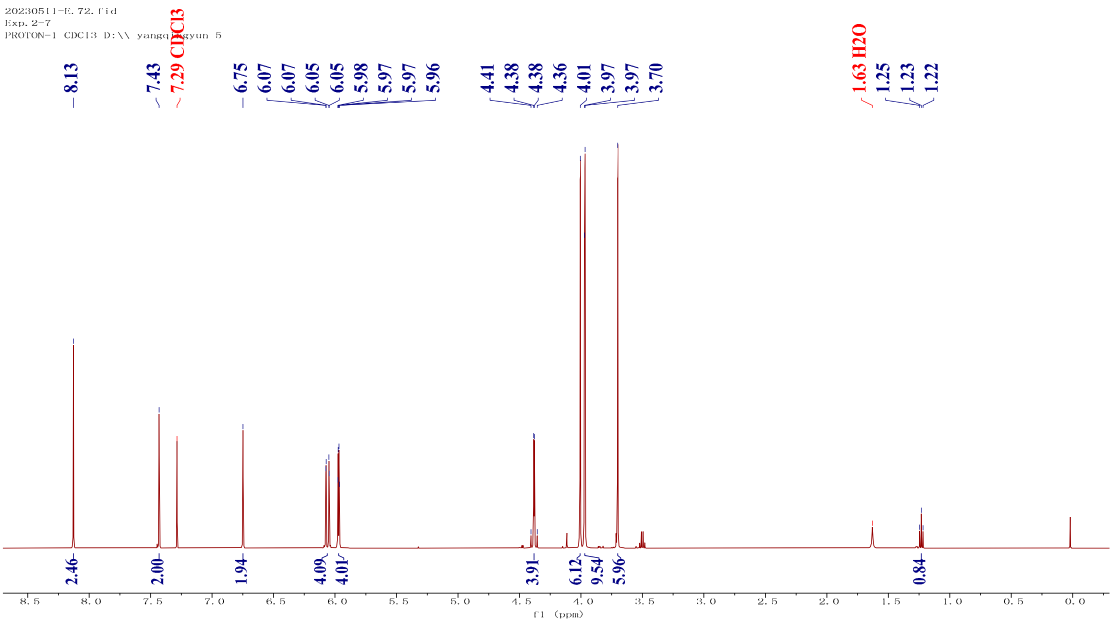

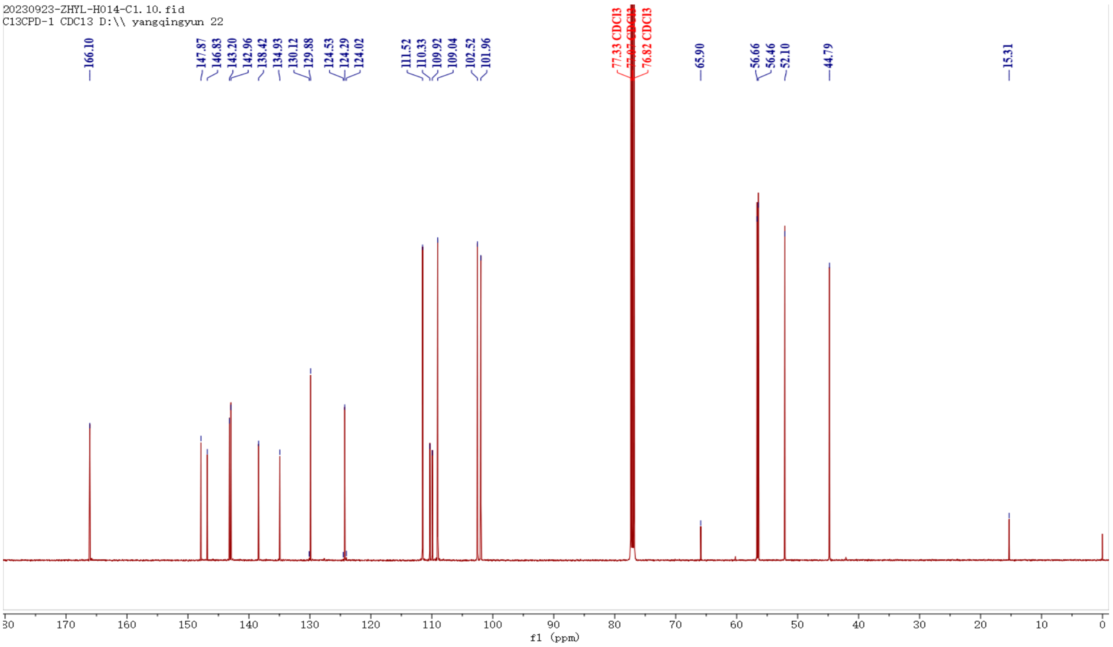

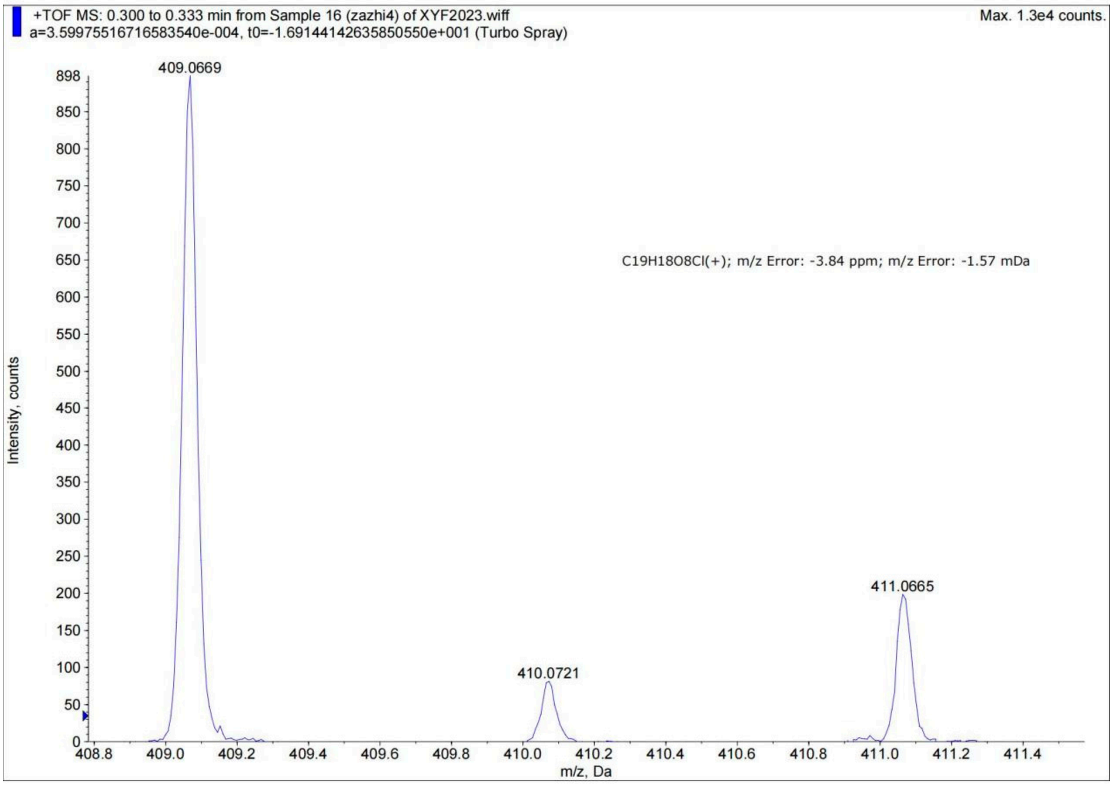

1H NMR (500 MHz, Chloroform-d) δ 8.13 (s, 2H), 7.43 (s, 2H), 6.75 (s, 2H), 6.06 (dd, J = 12.6, 1.4 Hz, 4H), 6.00 – 5.95 (m, 4H), 4.38 (d, J = 2.5 Hz, 4H), 4.01 (s, 6H), 3.97 (d, J = 2.3 Hz, 10H), 3.70 (s, 6H), 1.23 (t, J = 7.0 Hz, 1H). 13C NMR (126 MHz, CDCl3) δ 166.10, 147.87, 146.83, 143.20, 142.96, 138.42, 134.93, 130.12, 129.88, 124.53, 124.29, 124.02, 111.52, 110.33, 109.92, 109.04, 102.52, 101.96, 77.33, 77.07, 76.82, 65.90, 56.66, 56.46, 52.10, 44.79, 15.31. HR-ESI-MS: m/z [M+H]+ calcd for $C_{19}H_{18}ClO_8$ 409.0690, found 409.0669.

Figure S6. T1 relaxation time of quantitative signal of DMT, IMM-H014 and impurities I–IV

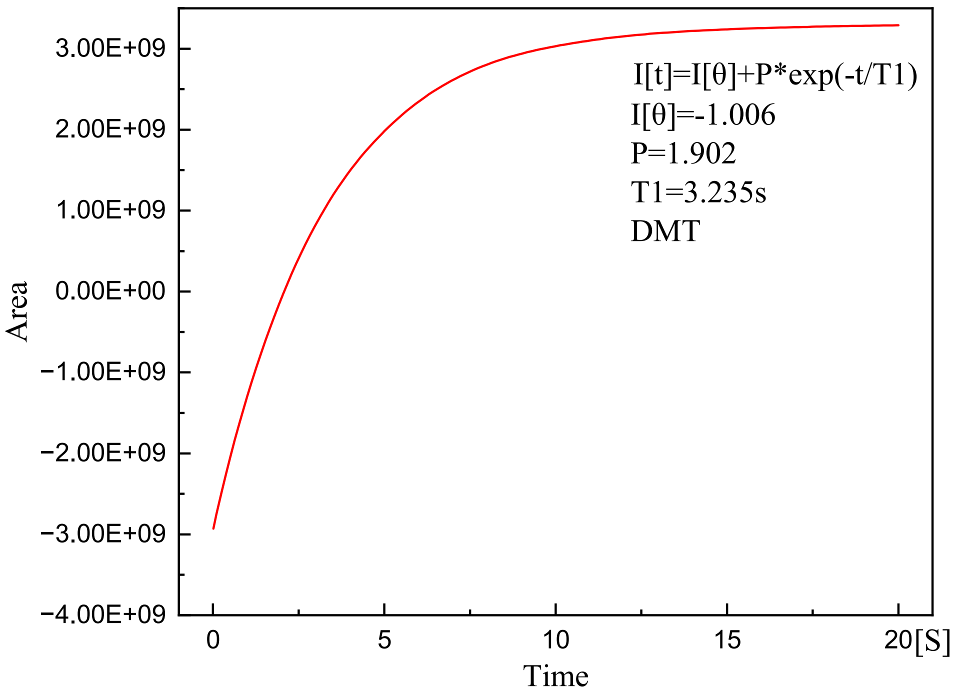

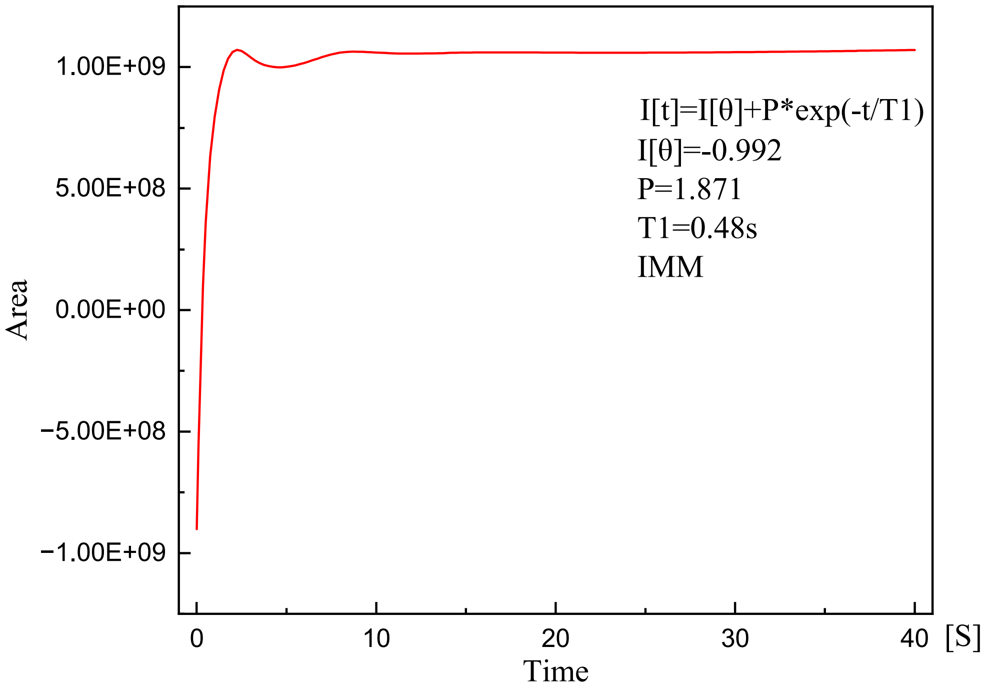

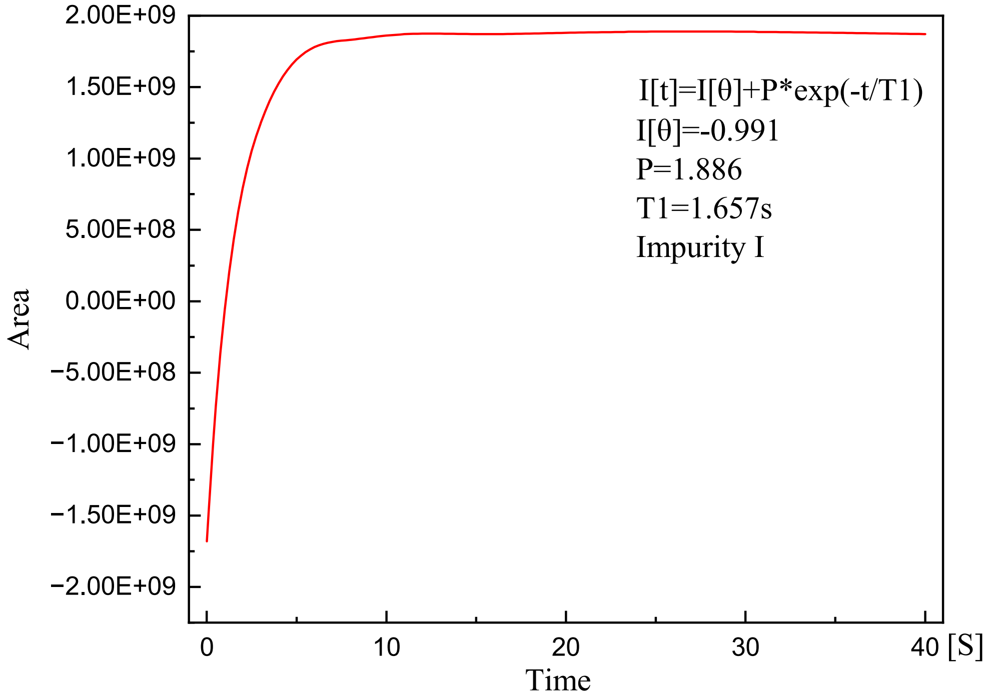
2.00E+09
1.50E+09
1.00E+09
5.00E+08
0.00E+00
−5.00E+08
−1.00E+09
−1.50E+09
−2.00E+09
Area
0
10
20
30
40
[S]
Time
I[t]=I[θ]+P*exp(-t/T1)
I[θ]=-0.991
P=1.886
T1=1.657s
Impurity I

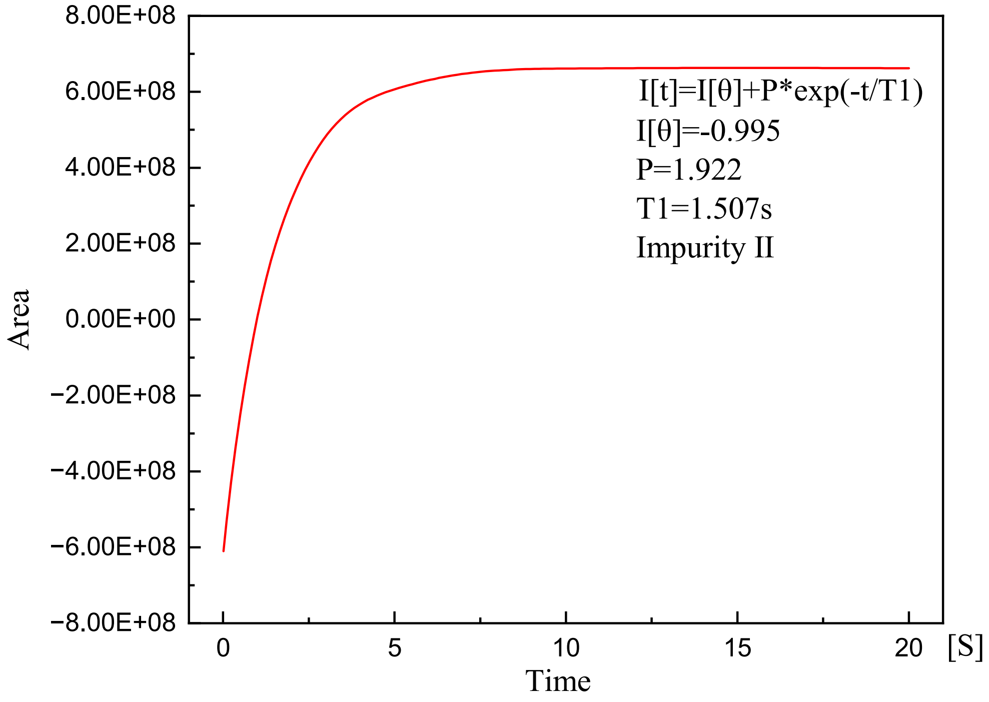
8.00E+08
6.00E+08
4.00E+08
2.00E+08
0.00E+00
−2.00E+08
−4.00E+08
−6.00E+08
−8.00E+08
Area
0
5
10
15
20
[S]
Time
I[t]=I[θ]+P*exp(-t/T1)
I[θ]=-0.995
P=1.922
T1=1.507s
Impurity II

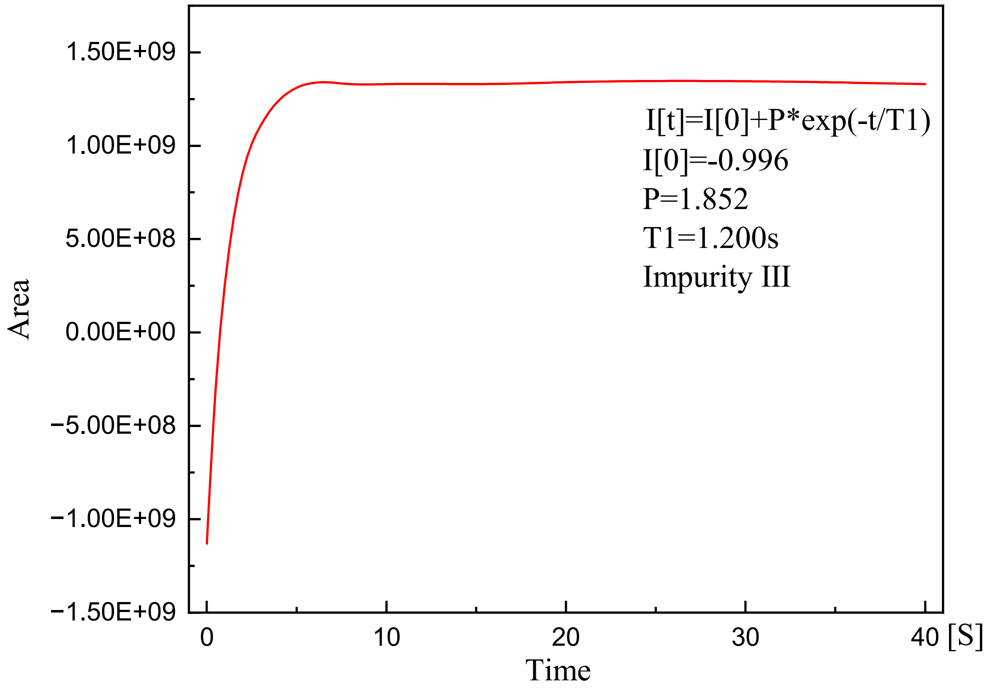
1.50E+09
1.00E+09
5.00E+08
0.00E+00
−5.00E+08
−1.00E+09
−1.50E+09
Area
0
10
20
30
40 [S]
Time
I[t]=I[0]+P*exp(-t/T1)
I[0]=-0.996
P=1.852
T1=1.200s
Impurity III

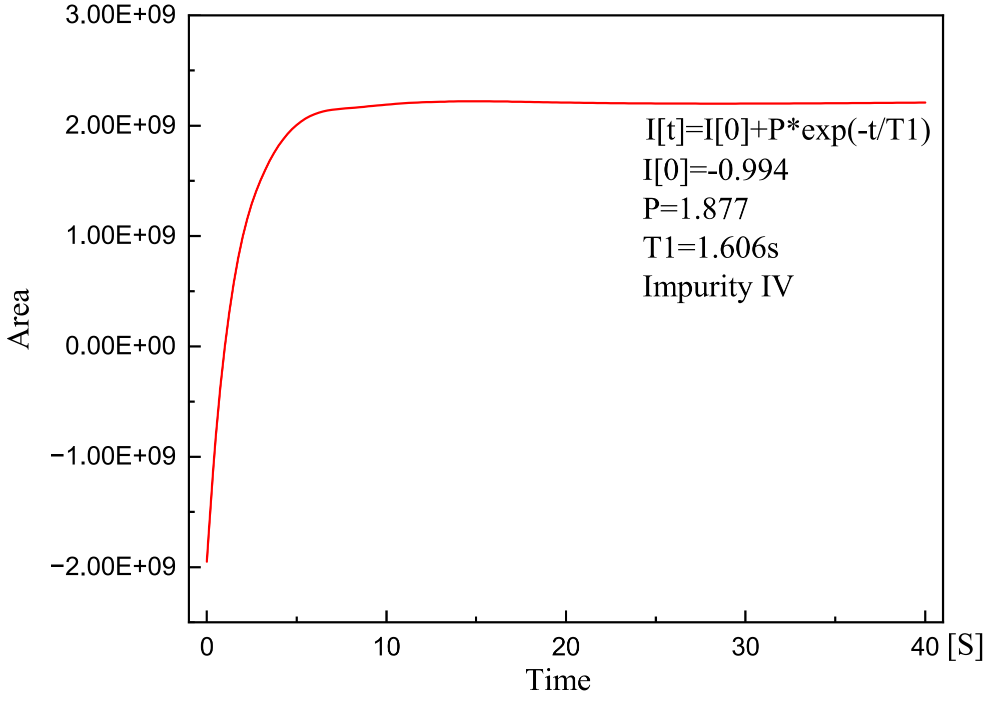
3.00E+09
2.00E+09
1.00E+09
0.00E+00
−1.00E+09
−2.00E+09
Area
0
10
20
30
40 [S]
Time
I[t]=I[0]+P*exp(-t/T1)
I[0]=-0.994
P=1.877
T1=1.606s
Impurity IV

Figure S7. Linearity calibration curves of IMM-H014 and its related substances

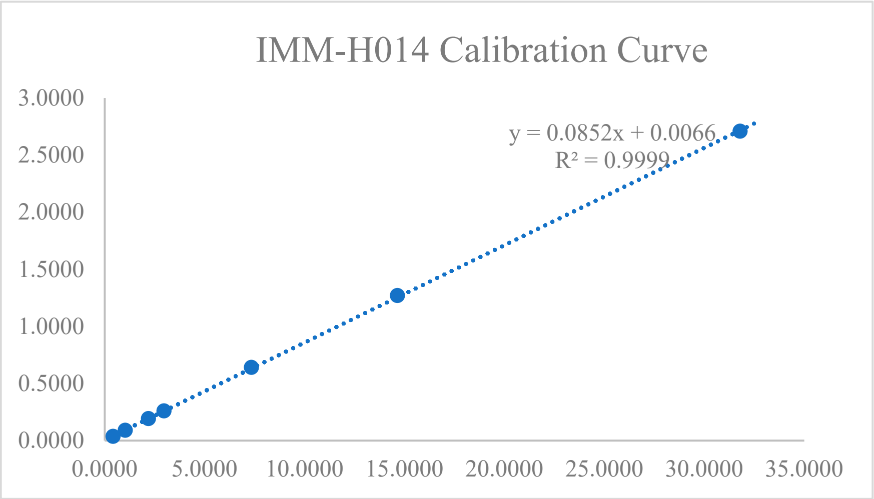

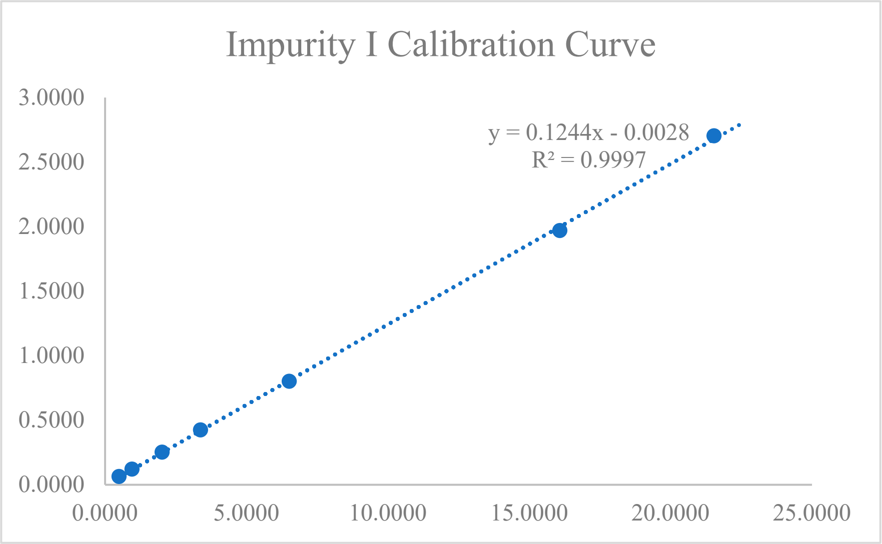

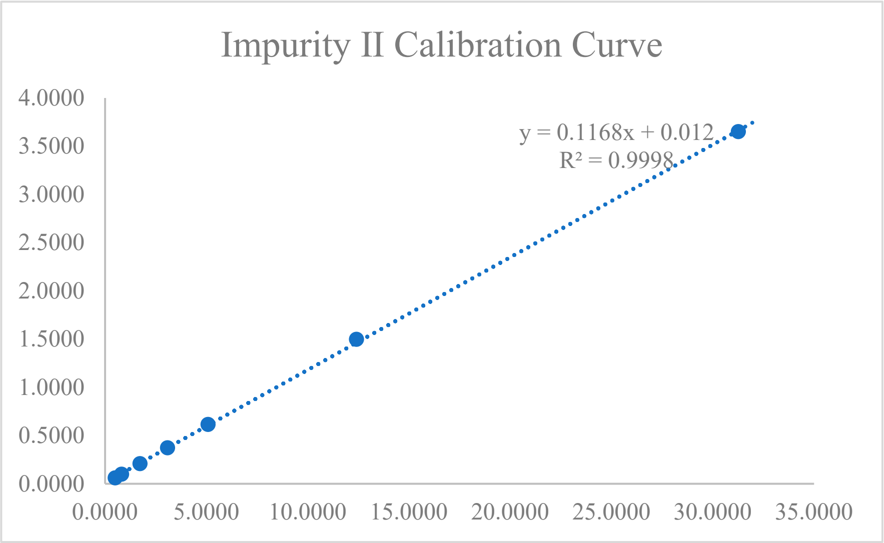

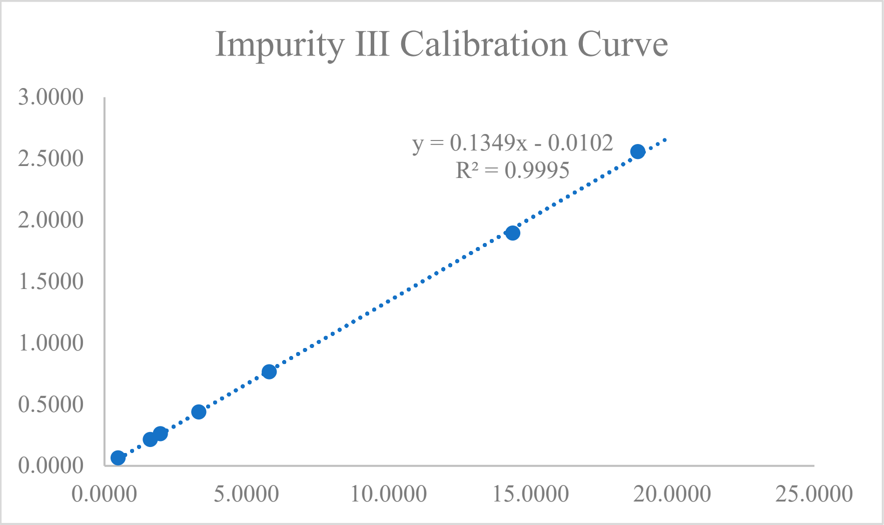

Impurity III Calibration Curve
y = 0.1349x - 0.0102
R² = 0.9995
3.0000
2.5000
2.0000
1.5000
1.0000
0.5000
0.0000
0.0000
5.0000
10.0000
15.0000
20.0000
25.0000

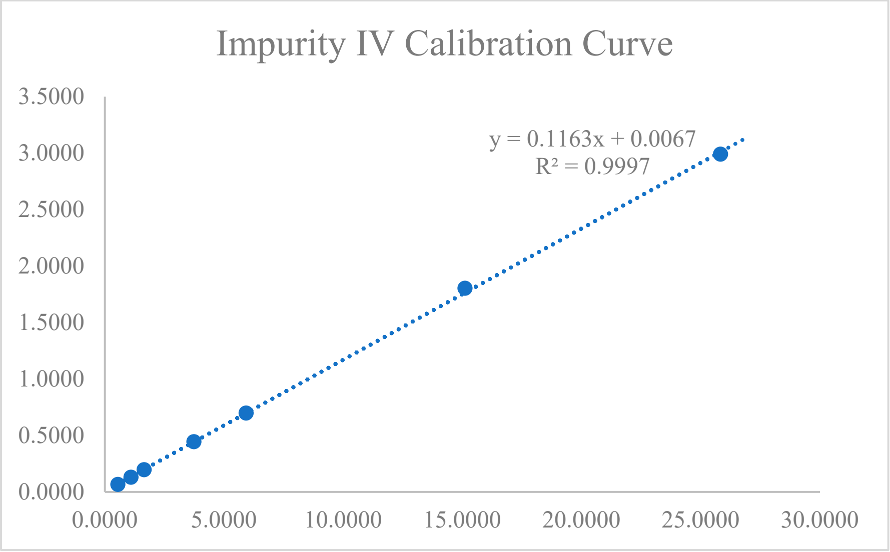

Impurity IV Calibration Curve
y = 0.1163x + 0.0067
R² = 0.9997
3.5000
3.0000
2.5000
2.0000
1.5000
1.0000
0.5000
0.0000
0.0000
5.0000
10.0000
15.0000
20.0000
25.0000
30.0000

Figure S8. TG test of IMM-H014 and Impurity I-IV

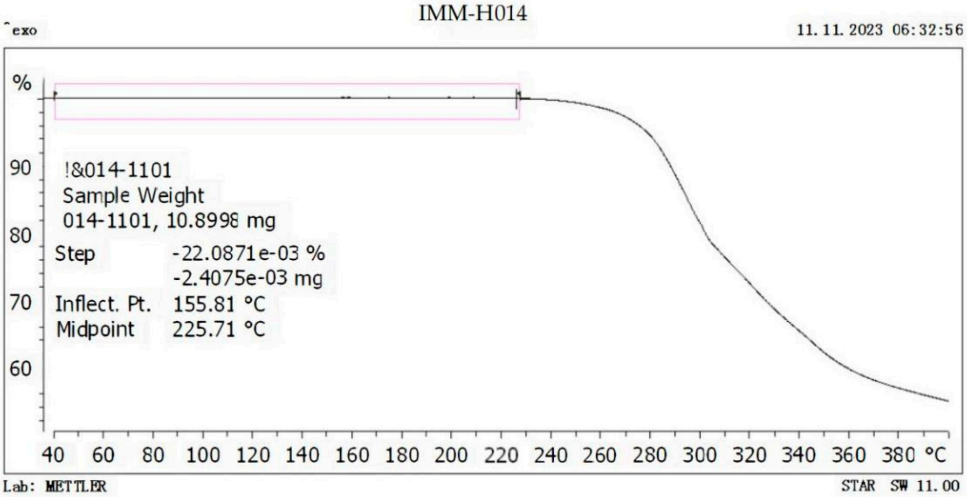

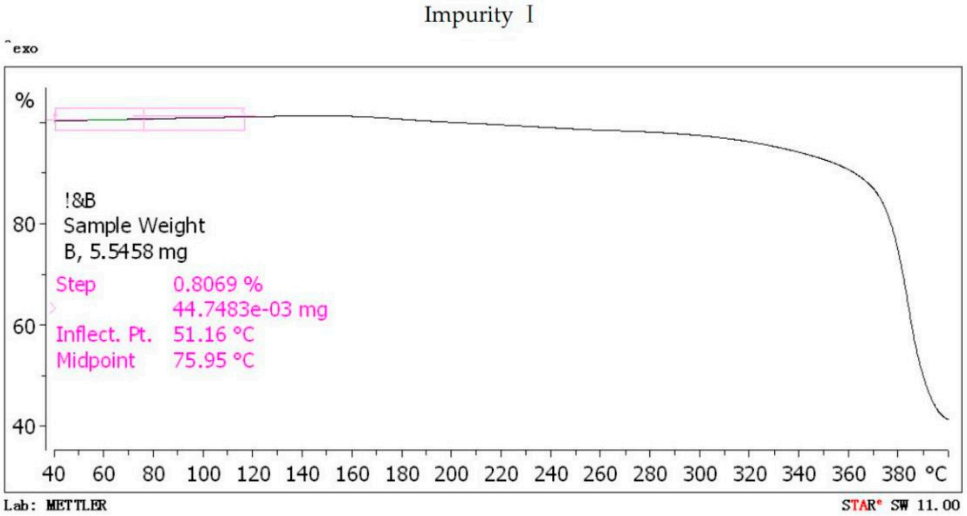

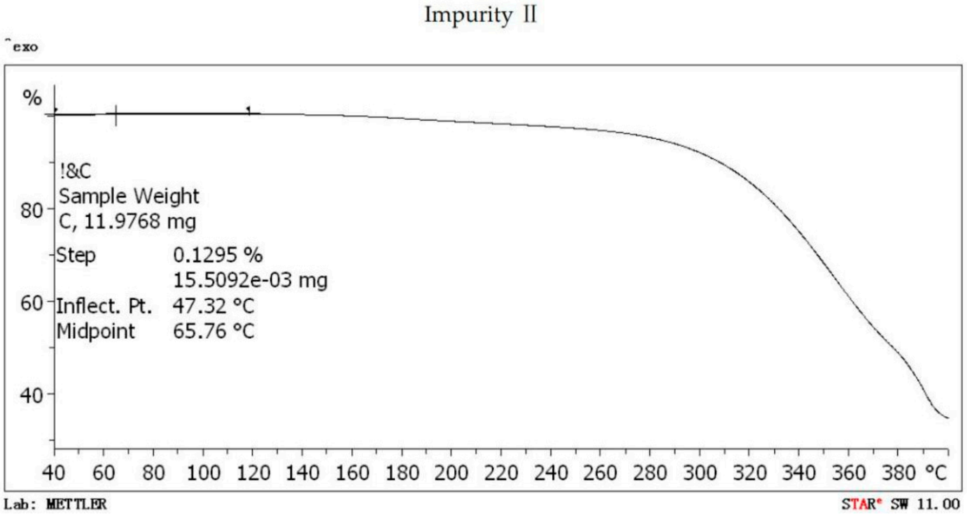

Impurity III

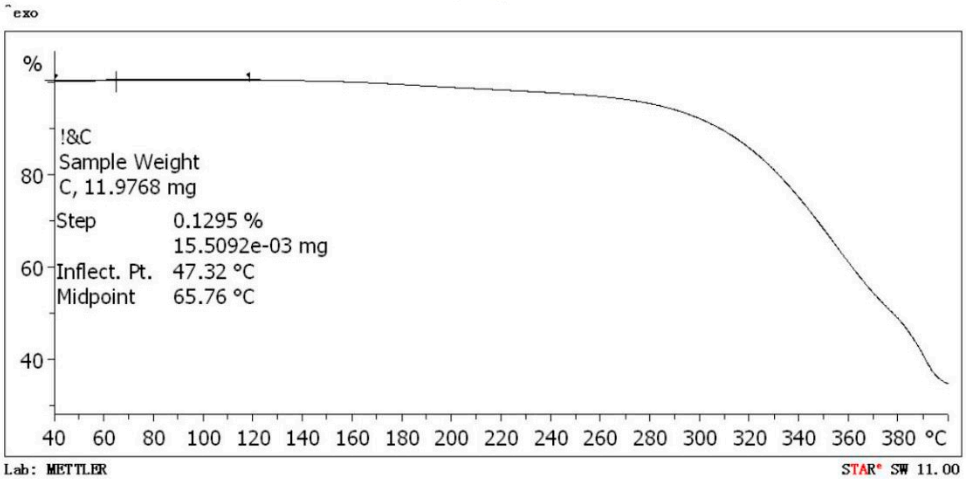

Impurity Ⅳ

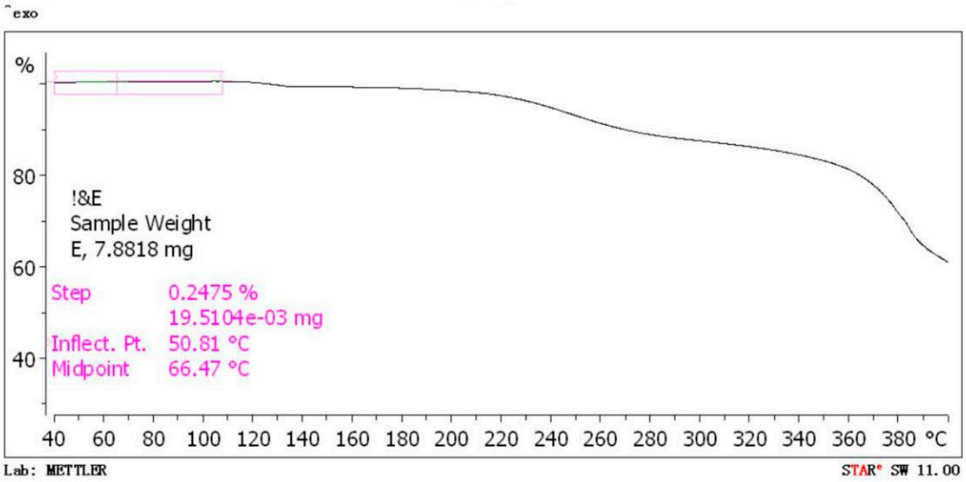

Table S1. Integration using different D1 value

D1/s	DMT	IMM-H014	Impurity I	Impurity II	Impurity III	Impurity IV
1	1.13	0.97	0.96	0.98	0.99	0.97
2	1.17	0.97	0.96	0.97	0.98	0.97
4	1.23	0.96	0.96	0.97	0.98	0.97
8	1.28	0.95	0.96	0.97	0.98	0.97
16	1.31	0.96	0.96	0.97	0.98	0.97
32	1.30	0.95	0.96	0.97	0.98	0.97
64	1.30	0.95	0.96	0.97	0.98	0.97

Table S2. Precision, accuracy and stability test of IMM-H014

	No.	mx	mstd	Px%	Average	RSD
Precision (n = 6)	1	18.138	2.469	99.27%		
	2	18.138	2.469	99.80%		
	3	18.138	2.469	99.61%	99.55%	0.23%
	4	18.138	2.469	99.61%		
	5	18.138	2.469	99.27%		
	6	18.138	2.469	99.76%		
Repeatability (n = 6)	1	18.138	2.469	99.67%		
	2	17.378	2.970	99.90%		
	3	16.686	2.097	99.85%	99.81%	0.38%
	4	16.223	2.755	99.60%		
	5	17.654	2.182	100.00%		
	6	17.129	3.507	99.82%		
Accuracy (recovery)	80%	16.223	2.755	99.82%		
		16.223	2.755	99.82%	99.82%	0.52%
		16.223	2.755	99.82%		
	100%	16.686	2.097	99.90%		
		16.686	2.097	99.60%	99.81%	0.19%
		16.686	2.097	99.82%		
	120%	17.129	3.507	99.67%		
		17.129	3.507	99.85%	99.82%	
		17.129	3.507	100.00%		0.61%
Stability	0h	18.138	2.469	99.27%		
	1h	18.138	2.469	99.80%		
	2h	18.138	2.469	99.61%		
	4h	18.138	2.469	99.61%		
	8h	18.138	2.469	99.27%	99.59%	0.20%
	12h	18.138	2.469	99.76%		
	24h	18.138	2.469	99.80%		
	36h	18.138	2.469	99.61%		
	48h	18.138	2.469	99.61%		

Table S3. Precision, accuracy and stability test of Impurity I

	No.	mx	mstd	Px%	Average	RSD
Precision (n=6)	1	8.673	1.330	99.20%	98.64%	0.44%
	2	8.673	1.330	98.27%		
	3	8.673	1.330	98.27%		
	4	8.673	1.330	98.46%		
	5	8.673	1.330	98.46%		
	6	8.673	1.330	99.20%		
Repeatability (n = 6)	1	8.673	1.330	98.58%	99.63%	0.70%
	2	8.17	1.686	99.33%		
	3	8.289	2.134	99.46%		
	4	8.697	1.119	99.86%		
	5	7.982	1.185	100.35%		
	6	7.917	1.313	100.17%		
Accuracy (recovery)	80%	6.402	1.208	99.62%	99.67%	0.59%
		6.402	1.208	99.71%		
		6.402	1.208	99.69%		
	100%	8.673	1.330	98.58%	98.54%	0.57%
		8.673	1.330	98.71%		
		8.673	1.330	98.34%		
	120%	10.153	1.286	100.53%	100.75%	0.33%
		10.153	1.286	100.86%		
		10.153	1.286	100.86%		
Stability	0h	8.673	1.330	99.20%	98.54%	0.57%
	1h	8.673	1.330	98.27%		
	2h	8.673	1.330	98.27%		
	4h	8.673	1.330	98.46%		
	8h	8.673	1.330	98.46%		
	12h	8.673	1.330	99.20%		
	24h	8.673	1.330	97.54%		
	36h	8.673	1.330	98.27%		
	48h	8.673	1.330	99.20%		

Table S4. Precision, accuracy and stability test of Impurity II

	No.	mx	mstd	Px%	Average	RSD
Precision (n=6)	1	8.459	1.662	100.27%	99.64%	1.15%
	2	8.459	1.662	101.22%		
	3	8.459	1.662	100.27%		
	4	8.459	1.662	99.33%		
	5	8.459	1.662	98.38%		
	6	8.459	1.662	98.38%		
Repeatability (n = 6)	1	8.459	1.662	100.59%	100.25%	0.91%
	2	8.170	1.686	99.49%		
	3	8.289	1.134	101.82%		
	4	8.697	1.119	99.52%		
	5	7.982	1.185	99.58%		
	6	7.917	1.313	100.51%		
Accuracy (recovery)	80%	6.625	1.127	100.59%	99.37%	1.02%
		6.625	1.127	98.70%		
		6.625	1.127	98.82%		
	100%	8.459	1.662	99.07%	99.33%	0.38%
		8.459	1.662	99.45%		
		8.459	1.662	99.48%		
	120%	9.704	1.206	99.14%	99.42%	0.92%
		9.704	1.206	99.44%		
		9.704	1.206	99.68%		
Stability	0h	8.459	1.662	100.27%	99.37%	1.02%
	1h	8.459	1.662	101.22%		
	2h	8.459	1.662	100.27%		
	4h	8.459	1.662	99.33%		
	8h	8.459	1.662	98.38%		
	12h	8.459	1.662	98.38%		
	24h	8.459	1.662	98.38%		
	36h	8.459	1.662	99.13%		
	48h	8.459	1.662	98.95%		

Table S5. Precision, accuracy and stability test of Impurity III

	No.	mx	mstd	Px%	Average	RSD
Precision (n=6)	1	7.553	1.300	97.64%	97.18%	0.44%
	2	7.553	1.300	97.42%		
	3	7.553	1.300	97.20%		
	4	7.553	1.300	97.42%		
	5	7.553	1.300	96.92%		
	6	7.553	1.300	96.47%		
Repeatability (n = 6)	1	7.553	1.300	97.42%	97.19%	0.33%
	2	7.553	1.940	97.00%		
	3	7.553	1.430	97.01%		
	4	7.553	1.941	97.16%		
	5	7.553	1.412	97.41%		
	6	7.553	1.681	97.17%		
Accuracy (recovery)	80%	7.553	1.942	97.16%	97.06	0.20%
		7.553	1.942	97.05%		
		7.553	1.942	96.97%		
	100%	7.553	1.300	97.42%	97.15%	0.37%
		7.553	1.300	96.94%		
		7.553	1.300	97.09%		
	120%	7.553	0.983	97.72%	97.55%	0.53%
		7.553	0.983	97.57%		
		7.553	0.983	97.37%		
Stability	0h	7.553	1.300	97.64%	97.15%	0.37%
	1h	7.553	1.300	97.42%		
	2h	7.553	1.300	97.20%		
	4h	7.553	1.300	97.42%		
	8h	7.553	1.300	96.92%		
	12h	7.553	1.300	96.47%		
	24h	7.553	1.300	97.36%		
	36h	7.553	1.300	96.99%		
	48h	7.553	1.300	96.92%		

Table S6. Precision, accuracy and stability test of Impurity IV

	No.	mx	mstd	Px%	Average	RSD
Precision (n=6)	1	8.215	1.384	98.49%	98.67%	0.33%
	2	8.215	1.384	99.12%		
	3	8.215	1.384	98.81%		
	4	8.215	1.384	98.49%		
	5	8.215	1.384	98.90%		
	6	8.215	1.384	98.21%		
Repeatability (n = 6)	1	8.215	1.384	98.81%	99.26%	0.67%
	2	7.908	1.018	99.58%		
	3	8.756	1.043	99.27%		
	4	8.320	1.643	99.97%		
	5	8.071	1.061	98.95%		
	6	8.605	1.044	98.99%		
Accuracy (recovery)	80%	6.726	1.572	99.79%	99.43%	0.56%
		6.726	1.572	99.54%		
		6.726	1.572	98.97%		
	100%	8.215	1.384	98.81%	98.94%	0.50%
		8.215	1.384	98.53%		
		8.215	1.384	99.48%		
	120%	9.576	0.973	99.31%	99.18%	0.80%
		9.576	0.973	98.70%		
		9.576	0.973	99.54%		
Stability	0h	8.215	1.384	98.49%	98.94%	0.50%
	1h	8.215	1.384	99.12%		
	2h	8.215	1.384	98.81%		
	4h	8.215	1.384	98.49%		
	8h	8.215	1.384	98.90%		
	12h	8.215	1.384	98.21%		
	24h	8.215	1.384	99.25%		
	36h	8.215	1.384	99.60%		
	48h	8.215	1.384	99.59%		
